# BACE2 tunes lipid uptake through lipid transporters shedding supporting cancer cell proliferation

**DOI:** 10.1186/s13046-025-03626-x

**Published:** 2026-01-08

**Authors:** Vittoria Matafora, Alice Elhagh, Alessandra Morelli, Laura Tronci, Angela Cattaneo, Camilla Conti, Francesca Casagrande, Francesco Farris, Angela Bachi

**Affiliations:** 1 IFOM ETS-The AIRC Institute of Molecular Oncology, Via Adamello 16, Milano, 16039 Italy; 2 Cogentech SRL Benefit Corporation, Milan, 20139 Italy; 3 SENTINEL CH S.p.a, Milan, Italy; 4 Present address: Human Technopole, Milan, Italy

**Keywords:** BACE2, Cancer, Lipid uptake, Lipid droplets, Lipidomics, Proteomics

## Abstract

**Background:**

Lipids play crucial roles in signal transduction, membrane integrity, and energy metabolism. In cancer, lipid metabolism is frequently dysregulated. Proliferating cancer cells exhibit enhanced lipid uptake, synthesis, and storage; however, the molecular mechanisms driving cancer metabolic reprogramming remain poorly understood. In this study, we identify Beta-secretase 2 (BACE2), a sheddase overexpressed in several solid tumors, as a critical regulator of lipid metabolism, revealing novel pathways underlying cancer metabolic vulnerability.

**Methods:**

We employed a multi-omics approach, including global and spatial proteomics, lipidomics and N-terminomics, combined with advanced imaging and functional assays to dissect BACE2’s role in lipid regulation and cancer metabolism. Metabolomic analyses and fluorescent lipid analog tracking were used to assess BACE2’s impact on lipid uptake. Finally, functional experiments elucidated the causal relationship between BACE2-dependent lipid regulation and tumor cells proliferation.

**Results:**

Analysis of patient tumor biopsies and cancer cell lines demonstrated a strong positive correlation between BACE2 expression, lipid metabolism, and lipid droplets (LDs) accumulation. Interestingly, extracellular lipid availability modulates BACE2 protein level, suggesting a feedback loop between lipid metabolism and BACE2 activity. Mechanistically, we proved that BACE2, assisted by the tetraspanin CD63, mediates the extracellular shedding of key lipid transporters, including the LDL receptor and the fatty acid transporter CD36, thereby tuning fatty acid and cholesterol uptake. BACE2 inhibition increased lipid influx, triggering the activation of PPAR-alpha signaling and LDs dynamics. In lipid-rich cancer cells this leads to lipolysis and metabolic stress, indicating that BACE2 activity is crucial for preventing lipolysis-induced damage. Inhibition of fatty acid uptake via CD36 blockade or suppression of ATGL-mediated lipolysis restores LDs integrity and rescues cell viability, underscoring BACE2’s role in maintaining a balanced lipid state critical for tumor cell survival and proliferation.

**Conclusions:**

Our findings uncover a novel function of BACE2 as a pivotal regulator of lipid transporter shedding, lipid uptake, and LDs homeostasis, ultimately shaping cancer cell metabolic adaptation and proliferation. These insights position BACE2 as a promising therapeutic target in lipid-addicted tumors, offering new avenues for cancer treatment.

**Supplementary Information:**

The online version contains supplementary material available at 10.1186/s13046-025-03626-x.

## Background

Lipids are fundamental components of biological membranes, key regulators of energy storage and metabolism, and critical signaling molecules in numerous cellular processes. The precise regulation of lipid metabolism, including uptake, synthesis, and hydrolysis, is critical for maintaining cellular homeostasis [1]. In cancer, lipid metabolism undergoes extensive reprogramming to support the high demands of tumor cells for rapid proliferation, survival, invasion, and metastasis formation. These metabolic alterations are now recognized as a hallmark of cancer [2], allowing malignant cells to adapt under diverse and often hostile conditions [3].

Among these metabolic changes, lipid uptake has emerged as a central adaptive mechanism in cancer cells, particularly within the tumor microenvironment (TME) [4]. As tumors grow and progress, they frequently experience hypoxia, fluctuating nutrient availability, and metabolic competition with surrounding immune and stromal cells [5]. To meet their metabolic needs, cancer cells increase lipid uptake, de novo lipogenesis, fatty acid oxidation (FAO), and lipid storage [5]. Overexpression of lipid uptake receptors, such as CD36 and the low-density lipoprotein receptor (LDLR), has been observed in various cancers, including lung, breast, ovarian, and pancreatic tumors, and is often correlated with poor clinical outcomes [6].

One prominent consequence of enhanced lipid uptake is the accumulation of lipid droplets (LDs), which is a well-established feature of many cancer types [7]. Beyond serving as storage sites for neutral lipids, LDs play protective roles by sequestering peroxidized lipids and damaged proteins, thus promoting cancer cell survival under metabolic and oxidative stress [8].

Despite clear evidence that enhanced lipid uptake promotes tumor progression, the underlying molecular mechanisms remain poorly defined. Various enzymes and regulatory proteins may influence lipid flux and utilization in cancer. In particular, lipid uptake is controlled by the surface availability of lipid transporters, which is dynamically regulated through endocytosis, recycling, degradation, and ectodomain shedding.

In this study, we identify BACE2 (Beta-Secretase 2), an aspartyl protease as a novel sheddase of lipid transporters, thereby controlling lipid uptake and homeostasis in cancer. Although initially characterized in the central nervous system alongside its homolog BACE1 for their roles in the generation of neurotoxic amyloid plaques in Alzheimer’s disease (AD) [9–11], BACE1 and BACE2 display distinct tissue distribution and physiological functions [12, 13].

BACE2 has been found involved in pancreatic β-cell maintenance [14], regulation of islet amyloid polypeptide (hIAPP) aggregation in type 2 diabetes (T2D) [15] and cleavage of VEGFR3 in lymphatic endothelial cells to modulate vascular signaling [16]. Its best-characterized role to date is the cleavage of PMEL, a melanocyte-specific protein, critical for eumelanin deposition [17].

Notably, BACE2 is upregulated in several solid tumors and correlates with cancer progression [9]. Elevated BACE2 expression has been reported in highly metastatic breast cancer cell lines [18], colon adenocarcinoma [19], metastatic melanoma [20] and pancreatic cancer [21]. Our group and others have shown that BACE2 expression correlates with highly proliferative cancer phenotypes, and its inhibition impairs tumor cell proliferation, migration, and invasion [21, 22]. Moreover, our previous work demonstrated that BACE2 activity modulates the extracellular deposition of amyloids aggregates [21] and promotes the secretion of various proteins, mainly involved in extracellular matrix (ECM) remodeling and lipid metabolism [20].

Here, using a multi-omics approach, we uncover a novel function for BACE2 in regulating lipid metabolism. Specifically, we show that BACE2 influences intracellular lipid homeostasis by modulating the processing of cholesterol and fatty acid transportes. These findings reveal a novel mechanism through which BACE2 contributes to cancer metabolic reprogramming, offering new insights into tumor adaptation and potential therapeutic vulnerabilities.

## Methods

### Reagents

The following reagents were obtained from the indicated suppliers: Standard SPLASH™ LIPIDOMIX™ Mass Spec, (Avanti Polar Lipids, cat. 330707);3I (Axon, cat. 1125); Atglistatin (Sigma-Aldrich, cat. SML1075); SSO(Sigma-Aldrich, cat. SML2148); DAPI (Sigma-Aldrich, cat. 28718-90-3); BODIPY FL C16 (Thermo Fisher Scientific, cat. D3821); 22-NBD-cholesterol (Thermo Fisher Scientific, cat. N1148); Nile Red (Thermo Fisher Scientific, cat. N1142); Filipin III (Sigma, cat. F4767); BODIPYTM 500/510 C1,C12 (4,4-Difluoro-5-Methyl-4-Bora-3a,4a-Diaza-s-Indacene-3-Dodecanoic Acid) (Thermo Fisher Scientific, cat. D3823); Lipid Mix (Chemically Defined Lipid Concentrate) (Gibco, cat. 11905031); DMSO (Euroclone, cat. EMR385258); Critical Commercial Assays MTT (3-(4,5-dimethylthiazol-2-yl)-2,5-diphenyltetrazolium bromide) (Sigma, cat. 1117140001); Mem-per plus kit (Thermo Scientific, cat. 89842); Bradford (Applied Biosystems, cat. 4368814).

### Antibodies

The following antibodies were purchased from the indicated suppliers: Pmel PEP13h (M. Marks donation); rabbit polyclonal anti-BACE2 (Sigma Prestige, cat. HPA035416); anti-Histone H3 (Abcam, cat. AB18521); anti E-cadherin (BD Biosciences, cat. 610181); anti-actin (Santa Cruz; cat. sc-47778); anti-cofilin (Cell Signalling, cat. 8503); rabbit polyclonal anti-CD36 (Novus Biologicals, cat. NB400144); CD63 mouse monoclonal antibody (Thermo Fisher Scientific, cat. 10628D); anti-LDLR mouse monoclonal antibody (1B10H10) (Thermo Fisher Scientific, cat. MA5-38556).

### Cell culture

Four melanoma cell lines (IGR39, IGR37 and WM266.4, WM115), PDAC cell lines (BXPC3, CAPAN-2), lung fibroblasts IMR90 and human embryonic Kidney HEK293T cells were used in this study. IGRs were purchased from DSMZ, WMs from Clinisciences, BXPC3 and HEK293T from ATCC, CAPAN-2 from IZSLER and IMR90 from CORIELL. Cancer cells and HEK293T were plated in 10 cm dishes and cultured in Dulbecco’s modified Eagle medium (DMEM) (Lonza) supplemented with 10% fetal bovine serum South American (FBS-SA, Euroclone) and 2 mM L-Glutamine (Euroclone). IMR90 cells were growth in MEM Complete Medium w/o Sodium Pyruvate containing non-essential amino acids 0.1mM, 1mM sodium pyruvate, supplemented with 10% fetal bovine serum South American (FBS-SA, Euroclone) and 2 mM L-Glutamine (Euroclone). Cells were incubated at 37 °C, 5% CO_2_. To mimic a lipids deprivation, cells were cultured in -DMEM, supplemented with 10% delipidated FBS (Biowest) and 2 mmol/ L-Glutamine. To mimic glucose deprivation, cells were cultured in -DMEM w/ glucose, supplemented with 10% dialyzed fetal bovine serum South American (FBS-SA, Euroclone) and 2 mM L-Glutamine (Euroclone). All of the cell lines were tested for mycoplasma, by mycoplasma PCR (Polymerase chain reaction) Test Kit.

### RNA extraction, RT–PCR and real-time PCR

Total RNA was extracted using Maxwell RSC simply RNA (Promega, USA) according to manufacturer’s instructions, and RNA was quantified by nanodrop. 1 µg of total RNA was used for retro-transcription using SuperScript™ VILO™cDNA Synthesis Kit (Invitrogen, USA). cDNA was diluted 1:10, and qPCR was performed using LightCycler^®^ 480 SYBR Green I Master (Roche, Switzerland). The primer sequences are provided below. Expression data were normalized to the geometric mean of the housekeeping gene RPLP0 to control the variability in expression levels and were analyzed using the 2‐ΔΔCT method.

Primers for qPCR:

**RPLP0**

Fw GTTGCTGGCCAATAAGGTG

Rv GGGCTGGCACAGTGACTT

**ACSL3**

Fw GGCGTAGCGGTTTTGACAC

Rv CCAGTCCTTCCCAACAACGA

**SCD1**

Fw TAGGGTAGGCTAGCTTAGTAG

Rv GCGTAGGGCATAGAAG

**CPT1**

Fw TAGAGACCAAACAAAGTAGATAGATAGATAGTCAG

Rv AGACGGTAGGAACAGAGGCTAGAAG

**SCARB1**

Fw GAGCTTTGGCCTTGGTCTACCT

Rv TCTTGTGCTCACTCCATTGTTTTG

**LRP8**

Fw CGGAACTATTCACGCCTCATC

Rv TCCTCTTTCGGGTCACTGG

**LDLR**

Fw AAGGACACAGCACACAACCA

Rv AAAGGAAGACCAGGAGCACG

### Immunofluorescence

#### Confocal image acquisition for lipid uptake analysis

Cells were plated on sterile 13 mm coverslips and when the cells were at 50% confluency, they were treated as indicated in the manuscript. One hour before fixation, cells were treated with BODIPY FL C16 (Thermo Fisher Scientific, D3821) 5 µM for FA uptake or with 22-NBD-cholesterol 4 µM to evaluate cholesterol uptake (Thermo Fisher Scientific, N1148), subsequently washed for three times with PBS and fixed with 4% (wt/vol) paraformaldehyde (PFA) for 10 min. BODIPY FL C16 and 22-NBD-cholesterol stock solutions were made in ethanol 100% at a concentration of 10 mM and 20 mM, respectively. For nuclei staining, fixed cells were incubated with DAPI (1:5,000) (Sigma–Aldrich D9542) for 3 min at room temperature, followed by three washes with PBS. Sample were analyzed using confocal microscopy performed using a Leica TCS SP5 or Leica TCS SP8 inverted microscopes, based on a Leica DMI 6000B or DMi8 respectively, and equipped with motorized stage. The images were acquired with an HCX PL APO 63X/1.4 oil immersion objective. We acquired BODIPY at 488 nm excitation and NBD-cholesterol at 470 nm excitation. The software used for all acquisitions was LAS AF or LAS X (Leica). Once the acquisition parameters for control conditions had been defined, they were kept constant for all the samples. The quantification of fluorescence intensity was performed using FiJi software (NIH).

#### Nile red staining

It was performed as previously reported [23]. Briefly, Nile red (Molecular Probes, Eugene, OR, USA) stock solution was made in DMSO at a concentration of 1 mg/mL. For cellular staining, cells were seeded on glasses coverslips and then fixed in PFA 4%, for 10 min. After washing, the cells were incubated for 1 h with Nile Red, 1:8000 Nile Red stock solution in 150 mM NaCl, protected from light. Nuclei were stained using DAPI, and finally, coverslips were mounted onto glass slides. Confocal microscopy was then performed using a Leica TCS SP5 inverted microscope mounted on a Leica DMI 6000B and equipped with motorized stage. The images were acquired with an HCX PL APO 63X/NA1.4 oil immersion objective using 488 nm laser line. Selectivity for lipid droplets is obtained when the cells were observed for yellow-gold fluorescence (excitation, 450–500 nm; emission, greater than 528 nm) rather than green fluorescence (excitation, 515–560 nm; emission, greater than 590 nm). To better appreciate the spheres, we showed the LD in green. The software used for all acquisitions was LAS AF (Leica). Lipid droplet quantification was performed using a Fiji.

#### Filipin staining

Cells were fixed with 4% paraformaldehyde in PBS for 10 min, washed three times with PBS, and stained with filipin III (Sigma F4767) 50 µg/mL in PBS for 45 min in darkness. Coverslips were mounted and filipin fluorescence was detected by fluorescence microscopy on Leica TCS SP8-STED inverted microscope based on a Leica DMi 8 and equipped with motorized stage. The images were acquired with an HCX PL APO 63X/NA1.4 oil immersion objective. The software used for all acquisitions Leica Application Suite X (ver. 3.5.2.18963). Filipin was acquired at 405 nm excitation.

#### Confocal live-cell imaging of BODIPY^™^ 500/510 C1,C12 loaded cells

BODIPY™ 500/510 (BODIPY™) was used to mark live cell lipid droplets. BODIPY™ is a fluorescent fatty acid analog that can be used as a marker of lipid droplets and membranes [24]. BODIPY^™^ 500/510 C1,C12 (4,4-Difluoro-5-Methyl-4-Bora-3a,4a-Diaza-s-Indacene-3-Dodecanoic Acid; Thermo Fisher Scientific, Molecular Probes™, catalog number: D3823) stock solution, 1 mg/mL, was prepared in ethanol and kept at − 20 °C until used. For time-lapses, the cells were seeded in IBIDI chambers on a glass slide suited for confocal analysis. They were treated as showed in the manuscript. One hour before imaging, 1 µg/mL BODIPY™ was dissolved in the medium, then cells were washed with PBS and rinsed with medium for the acquisition. Confocal imaging was performed using an Olympus CSU spinning disk system mounted on an IX83 inverted microscope equipped with a motorized stage and an Andor iXon Ultra 897 camera (16 bit, 16 μm pixel size). We acquired BODIPY using a UPlanSApo 20X/0.75 dry objective and 488 nm laser line. The software used for the acquisitions was CellSens Dimension (Olympus).

### BACE2 overexpression

For BACE2 overexpression plasmid constructs for BACE2-Flag and FLAG-empty vector gifted by Guillaume Van Niel group were used. Briefly, HEK293T cells were plated to reach 50% confluency on the day of transfection. For each transfection, two sterile polypropylene tubes were used: Tube A contained 500 µL of 2× HBS; Tube B contained 439 µL of sterile distilled H₂O, 10 µg of plasmid DNA (EV or BACE2), and 61 µL of 2 M CaCl₂. While bubbling in Tube A continuously, the contents of Tube B were added dropwise. The mixture was incubated at room temperature for no longer than 5 min to allow precipitate formation. The resulting DNA/CaPO₄ suspension was added directly to the cells. Cells were incubated for 16 h then the transfection medium was replaced with fresh culture medium. 72 h hours after transfection, cells were harvested for further analysis.

### Proteomics analysis

#### Secretome Preparation from cell cultures

Secretome analysis was performed as showed previously [25]. Briefly, Cells were grown in a DMEM except for IMR90 which was grown in MEM medium. Cells were counted and equal numbers of cells were seeded into 10-cm dishes at roughly 50% confluence. Once cell lines reached ~ 70% confluence, cells were treated as indicated in the manuscript and were starved in serum‐free media for 24 h. The conditioned media (CM) were centrifuged (800 g, 3 min), filtered (0.22 μm) to remove detached cells, and concentrated via centrifugation at 4,500 g in 10 kDa molecular weight cutoff concentrating columns. Then, 500 µl of concentrated medium was filtered by microcon filters with 10 kDa cutoff (Millipore) and buffer was exchanged with 8 M Urea 100 mM Tris or PBS. Proteins secreted by 2 × 10^6^ cells were replaced in 8 M Urea 100 mM Tris HCl pH 8 and sonicated with BIORUPTOR (3 cycles: 30 s on/30 s off). By using microcon filters with 10 kDa cutoff (Millipore), cysteine reduction and alkylation were performed adding 10 mM Tris(2-carboxyethyl)phosphine hydrochloride (TCEP) (Thermo Scientific) and 40 mM 2‐Chloroacetamide (CAM) (Sigma‐Aldrich) in 8 M Urea 100 mM Tris HCl pH 8 for 30 min at room temperature, as described for the FASP protocol [26]. Buffer was exchanged by centrifugation at 9,300 g for 10 min, and PNGase F (New England Biolabs) (1:100 = enzyme: secreted proteins) was added for 1 h at room temperature following manufacturer’s instruction. Buffer was again exchanged by centrifugation at 9,300 g for 10 min with 50 mM ammonium bicarbonate, and proteins were in solution digested with trypsin (Trypsin, Sequencing Grade, modified from ROCHE) (1:50 = enzyme: secreted proteins) overnight at 37 °C. Peptides were recovered on the bottom of the microcon filters by centrifugation at 9,300 g for 10 min and on the top, adding two consecutive washes of 50 µl of 0.5 M NaCl. Eluted peptides were purified on a C18 StageTip. 1 µg of digested sample was injected onto a quadrupole Orbitrap Q‐exactive HF mass spectrometer (Thermo Scientific) Peptide separation was achieved on a linear gradient from 95% solvent A (2% ACN, 0.1% formic acid) to 55% solvent B (80% ACN, 0.1% formic acid) over 75 min and from 55% to 100% solvent B in 3 min at a constant flow rate of 0.25 µl/min on UHPLC Easy‐nLC 1000 (Thermo Scientific) where the LC system was connected to a 23‐cm fused‐silica emitter of 75 μm inner diameter (New Objective, Inc. Woburn, MA, USA), packed in‐house with ReproSil‐Pur C18‐AQ 1.9 μm beads (Dr Maisch Gmbh, Ammerbuch, Germany) using a high‐pressure bomb loader (Proxeon, Odense, Denmark).

The mass spectrometer was operated in DDA mode as described previously: [27] dynamic exclusion enabled (exclusion duration = 15 s), MS1 resolution = 70,000, MS1 automatic gain control target = 3 × 10^6^, MS1 maximum fill time = 60 ms, MS2 resolution = 17,500, MS2 automatic gain control target = 1 × 10^5^, MS2 maximum fill time = 60 ms, and MS2 normalized collision energy = 25. For each cycle, one full MS1 scan range = 300–1,650 m/z was followed by 12 MS2 scans using an isolation window of 2.0 m/z.

#### MS analysis and database search

MS analysis was performed as reported previously [27]. Raw MS files were processed with MaxQuant software (1.5.2.8), making use of the Andromeda search engine [28] MS/MS peak lists were searched against the UniProtKB Human complete proteome database uniprot_cp_human_2015_03) in which trypsin specificity was used with up to two missed cleavages allowed. Searches were performed selecting alkylation of cysteine by carbamidomethylation as fixed modification, and oxidation of methionine, N-terminal acetylation and N‐Deamination as variable modifications. Mass tolerance was set to 5 ppm and 10 ppm for parent and fragment ions, respectively. A reverse decoy database was generated within Andromeda, and the false discovery rate (FDR) was set to < 0.01 for peptide spectrum matches (PSMs). For identification, at least two peptide identifications per protein were required, of which at least one peptide had to be unique to the protein group.

#### Membrane proteome

Membrane proteome preparation was performed using Mem-per plus kit (Thermo Scientific™, 89842), according to manufacturer’s protocol. The cells were first permeabilized with a mild detergent, allowing the release of soluble cytosolic proteins, after which a second detergent solubilizes membrane protein. Briefly, cells were washed with PBS and centrifuged at 900 x g for 5 min. The supernatant was removed, the cells were resuspended in resuspended in 400 µL of permeabilization Buffer, briefly vortexed, and incubated for 10 min at 4 °C on rotation. The samples were then centrifuged at 16,000 x g for 15 min. The supernatant containing the cytosolic proteins was removed, and 0.2 mL of solubilization buffer was added to each tube. The samples were then incubated at 4˚C on rotation for 30 min, followed by centrifuging at 16,000 x g for 15 min at 4˚C. The supernatant containing the membrane protein fraction was then digested for LCMSMS analysis.

#### Global proteome

Cells were counted and equal numbers of cells were seeded in 10-cm dishes at roughly 50% confluence. Once cell lines reached ~ 70% confluence, cells were treated as indicated in the manuscript. At the end of the treatments, cells were pelleted and resuspended with Urea 8 M, Tris HCl 150mM pH8. UA buffer. Proteins were quantified by BCA assay (Thermo scientific). 50ug of proteins were digested for LCMSMS. Briefly, Cysteine reduction and alkylation were performed in 10 mM TCEP (Thermo scientific) and 40 mM 2‐CAM (Sigma‐Aldrich) in 8 M Urea 100 mM Tris HCl pH 8 for 30 min at room temperature. Protein in-solution digestion was performed by incubating each sample in 100 mM Tris HCl ph8, 6 M Urea supplemented with LysC 1:30 w/w; and 1 h later supplemented with trypsin 1:50 w/w in 100 mM Tris HCl pH 8, 2 M Urea overnight at 25 °C. The obtained peptides were collected, purified on a C18 StageTip (Proxeon Biosystems) and split in two independent samples for technical replicates.

#### Global and membrane proteomics MS analysis

1.5 µl of digested sample was injected onto an Exploris 480 mass spectrometer (Thermo Scientific) equipped with FAIMS device (Thermo Scientific). Columns were packed in-house with ReproSil-Pur C18-AQ beads (Dr. Maisch Gmbh, Ammerbuch, Germany), 1.9 μm of diameter, using a high-pressure bomb loader (Proxeon, Odense, Denmark).

Peptides separation was achieved with a linear gradient from 95% solvent A (2% ACN, 0.1% formic acid) to 41% solvent B (80% ACN, 0.1% formic acid) over 124 min and from 41% to 100% solvent B in 9 min at a constant flow rate of 0.25 µL/min, with a single run time of 131 min. The mass spectrometer was operated in ESI + in data-dependent acquisition (DDA) mode or data dependent analysis (DIA).

Parameters for (DDA) mode: charge state: 2–6, intensity threshold 5.0 × 10^3^, dynamic exclusion enabled (exclusion duration = 20 s), MS1 resolution = 60,000, MS1 automatic gain control target = 1 × 10^6^ (100%), MS1 maximum injection time = 100 ms, MS2 resolution = 15,000, MS2 automatic gain control target = 1 × 10^6^ (100%), MS2 maximum fill time = Auto, and MS2 HCD collision energy % = 28. For each cycle, one full MS1 scan range = 300–1,500 m/z was followed by 28 MS2 scans using an isolation window of 1.6 m/z.

Parameters for (DIA) mode: charge state: 2–6, MS1 resolution = 120,000, precursor range 400–1000 m/z, MS1 automatic gain control target = 3 × 10^6^ (300%), MS1 maximum injection time = 55 ms, MS2 resolution = 30,000, MS2 automatic gain control target = 10 × 10^6^ (1000%), MS2 maximum fill time = Auto, Radio Frequency lens 40%, and MS2 HCD collision energy % = 32, 30 m/z isolation windows. the MS2 scan range was Auto, and the loop control was set to All.

The FAIMS device was operated with the following compensation voltages: −50 V and − 70 V with a total carrier gas flow of 3.7 L/min at 300 °C for Ion transfer tube temperature.

#### DDA MS analysis and database search

Raw MS files were converted in MzxML files with FAIMS-MzxML generator (https://github.com/coongroup/FAIMS-MzXML-Generator) as they contain multiple CVs and were analyzed with MaxQuant (version 1.6.0.16), using Andromeda as searching engine.

MS/MS peak lists were searched against the UniProtKB Human complete proteome database (uniprot_cp_human_2020) in which trypsin specificity was used with up to two missed cleavages allowed. Searches were performed selecting alkylation of cysteine by carbamidomethylation as fixed modification, and oxidation of methionine, N-terminal acetylation and N-Deamination as variable modifications.

Mass tolerance was set to 5 ppm and 10 ppm for parent and fragment ions, respectively. A reverse decoy database was generated within Andromeda, and the false discovery rate (FDR) was set to < 0.01 for peptide spectrum matches (PSMs). For identification, at least two peptide identifications per protein were required, of which at least one peptide had to be unique to the protein group.

#### DIA MS analysis and database search

The DIA raw files of FAIMS-DIA were searched by Spectronaut (SN) vs. 18. These searches were directly used by Pulsar (a search engine embedded in SN) to generate DDA-based spectral libraries via the “Generate Library from Pulsar/Search Archives” function. The default BGS factory settings, using UniProt cp. Human proteome reference, were used when generating all libraries. For DIA searches comparison, Trypsin was selected as the digestion enzyme. Carbamidomethylation was selected as a fixed modification, while methionine oxidation and N-terminal acetylation were selected as variable modifications. FDRs of PSMs and peptide/protein groups were set to 0.01. “Area” MS1 quantity type was used with Cross-Run Normalization “ON”.

#### N-TAILS

For degradomics analysis of protein substrates, the proteomic method TAILS was used to isolate and quantify cleaved neo-N-terminal peptides [29]. Briefly, IGR37 and WM266.4 cells were grown in DMEM, 10% FBS to 50% confluency. Cells were treated with 3I or DMSO as control for 24 h. Cells were washed and treated with 3I or DMSO as control in serum-free medium for additional 24 h. Conditioned medium proteins were clarified by centrifugation (5 min, 900 × *g*) and filtration (0.22 μm). The proteins were concentrated by ultrafiltration using Vivaspin^®^ 20, 10 kDa MWCO Polyethersulfone to ˜ 100 µl. The sample buffer was exchanged to HEPES 0.1 M. Protein concentration was determined by BCA assay (Pierce, Rockford, IL). Proteins were reduce and alkylated 30 min with TCEP and 2- CAM, then N-terminus amines of respectively DMSO and 3I proteins were differentially labelled with light and heavy formaldehyde. The reaction occurred at 37 °C overnight and was catalyzed by NaBH_3_CN. The day after fresh formaldehyde was added for 2 h more and reaction was quenched 1 h with Tris HCl 0.1 M pH6.8. DMSO and 3I samples were pooled together for each replicate, then proteins were precipitated and resuspended in buffer GlycoBuffer 1X where they were digested at 37 °C for3/4 h by PNGaseF. Buffer was changed to HEPES 0.1 M and GuHCl 3 M, using Amicon Ultra centrifugal filter units (10 kDa cutoff, Millipore). Proteins were digested at 37 °C overnight by trypsin and the day after N-terminus peptides were negative selected by adding the HPG-ALD polymers the only react with trypsin-generated N-termini. Reaction occurred at 37 °C overnight and following N-terminome was recovered in the flowthrough after using Amicon Ultra centrifugal filter units (10 kDa cutoff, Millipore). Desalted peptides were analyzed following liquid chromatography in DDA mode, with a gradient of 131 min. The following parameters were applied to MaxQuant serch for TAILS peptides: Oxidation of Methionine, Acetylation of N-termini and pyro-Glus as variable modification, Carbamidomethylation of cysteins as fixed modification, DimethLys and DimethNterm as fixed labels and semi-ArgC cleavage specificity with up to two missed cleavages. Peptide annotation and statistical analysis were performed with MANTI (https://pubs.acs.org/doi/10.1021/acs.analchem.1c00310).

### Western blot

For Western blot analyses, proteins were extracted in buffer containing 8 M Urea, 100 mM Tris HCl pH 8. Briefly, cell lysates (50 µg) were separated by SDS–PAGE using a precast polyacrylamide gel with a 4% to 12% gradient (Invitrogen). After the electrophoretic run, proteins were transferred onto a 0.22 μm nitrocellulose membrane, for dot blot we spotted the protein mix directly to nitrocellulose membrane (Amersham Protran, GE Healthcare) in wet conditions. The assembled sandwich was loaded in a Trans-Blot Cell (Bio‐Rad) and immersed in 1× cold Tris‐Glycine transfer buffer with the addition of 20% methanol. The transfer was allowed overnight at constant voltage (30 V). Correct protein transfer was verified staining the membrane with Ponceau red (Sigma‐Aldrich) for few seconds. After washing the membrane with Tris‐buffered Saline‐Tween 20 (TBST, 1× TBS with 0.1% Tween‐20), non‐specific binding of antibodies was blocked by adding 5% low‐fat dry milk in TBST for 1 h at room temperature. The antibody used are anti-BACE2 (Sigma Prestige, 1:250, rabbit) anti-actin.

### BACE2 and CD63 Silencing

IGR39 and IGR37 cells were transfected using ScreenFect siRNA reagent according to manufacturer’s instruction. Briefly, two mixtures were prepared; for MixA, 4 µL ScreenFect siRNA were diluted in 120 µL of ScreenFect dilution buffer, and for MixB, 10 nM of siRNA against non-coding region (siNC) or siRNA against BACE2 (siBACE2) or siRNA against CD63 (siCD63) were diluted in 120 µL of ScreenFectA dilution buffer. MixA and MixB were then mixed together using rapid, slight pipette strokes and incubated for 20 min at room temperature to allow siRNA/Lipofectamine complex formation. After that, 1260 µL fresh cell suspension at a concentration of 2 × 10^5^ cells/mL were added to the complexes and gently mixed with a pipette and plated in 6-well plates.

The following day, the cell culture medium was changed to avoid the toxic effects of lipofectamine. Cells were collected after 48 h to assess the silencing and to perform downstream experiments. Sequences of siRNA used:

**Table:** 

siBACE2	GAUUCUCGUUGACACUGGA UCCAGUGUCAACGAGAAUC
siCD63	GUU CUU GCU CUA CGU CCU C
siNC	ACGUGACACGUUCGGAGAA UUCUCCGAACGUGUCACGU

### MTT cell viability assay

To perform 3-(4,5-dimethylthiazol-2-yl)-2,5-diphenyltetrazolium bromide (MTT; Sigma) cell viability assay, melanoma cells were seeded in 96-well plates and were treated with 3I, rPMEL amyloid fibrils or combination of the two, as indicated in the text. At the end of the experiments, the cell cultures were supplemented with 150 µl of 0.5 mg/mL MTT assay and incubated for an additional 4 h. Then, equal volume of solubilizing solution (dimethyl sulfoxide 40%, SDS 10% and acetic acid 2%) was added to the cell culture to dissolve the formazan crystals and incubated for 10 min at room temperature. The absorbance rate of the cell culture was detected at 570 nm by using a Microplate Reader (Bio-Rad, Hercules, CA, USA).

### Statistical analysis

All statistical analysis of the experimental data was performed using the GraphPad Prism version 9.5.0 for macOS, GraphPad Software, San Diego, CA, USA, www.graphpad.comsoftware. All the experiments were performed using at least 3 biological replicates. Statistical significance for each experiment is marked in the form of asterisks (*) along with the calculated *p*-value for experiments are shown.

### Lipidomics

#### Lipids extraction (extra-intra lipidome)

Melanoma cells were seeded in six-well tissue culture plates at 300,000 cells per well in 2 ml of DMEM. The medium alone (2 ml) was placed in an additional well to serve as a medium control. Cells and control medium were maintained in culture at 37 °C, 5% CO_2_. The day after seeding, cells were treated as indicated in the manuscript. At the time of sample collection, the medium was taken from each well, snap-frozen on dry ice, and stored at − 80 °C for processing and analysis of the extracellular lipid profile. Medium samples were thawed. To remove proteins, 400 µl from each sample was filtered through a 3-kDa cutoff micron filter (Millipore) by centrifugation for 20 min at 13,000*g*. Filtered medium was divided in two aliquots; 170 µl of each aliquot was spiked with 0.5 µl of SPLASH LIPIDOMIX Mass Spec Standard. Lipid extraction was performed by adding 700 µl of methanol to each sample, followed by sonication for 1 min at 4 °C. Next, 350 µl of chloroform was added to each sample. Samples were mixed on an orbital shaker for 15 min at 4 °C. Then, 350 µl of water/chloroform (1:1, v/v) was added to each sample. The samples were then centrifuged at 13,000*g* for 10 min at 4 °C. The lower phase of each sample was carefully isolated and dried in a SpeedVac before being resuspended and pooled with the appropriate paired aliquot in 25 µl of a buffer composed of 10% of ethanol and 90% of buffer made of 95% mobile phase A (ACN: water 40:60; 5 mM ammonium acetate; 0.1% formic acid) and 5% mobile phase B (isopropanol: water 90:10; 5 mM ammonium acetate; 0.1% formic acid). For the assessment of intracellular lipidomes, cells were harvested via trypsinization. Culture medium was used to inactivate the trypsin. The cell pellet was washed twice with 1× PBS before being resuspended in 200 µl of cold MilliQ H2O. Lipids were extracted and processed using a single-step extraction protocol with methanol and chloroform as described previously [30]. In brief, cells were lysed by passing them repeatedly through a 26-gauge needle. 20 µl of sample was added to 5 µl of 5× lysis buffer containing 10% NP-40 and 2% SDS in 1× PBS. Following a 30-min incubation on ice, the samples were centrifuged at 13,000*g* for 20 min at 4 °C. The clarified lysates were quantified using BCA assay. An equivalent of 50 µg of protein per sample was then used for the lipid extraction. In brief, the resuspended pellets were supplemented with cold water to obtain a final volume of 170 µl per sample. Each sample was spiked with 1 µl of SPLASH LIPIDOMIX Mass Spec Standard and then 700 µl of methanol was added. Samples were sonicated using Bioruptor for 60 s at 4 °C. Cold chloroform (350 µl) was added. Samples were vortexed and incubated on an orbital shaker for 15 min at 4 °C. In total, 350 µl of a 1:1 chloroform: water mixture was then added to each sample. Next, samples were vortexed and centrifuged at 13,000*g* for 10 min at 4 °C. The lower phase was carefully isolated and dried in a SpeedVac before being resuspended as previously described.

#### Lipidomics MS analysis and database search

One µl of extracted lipids was diluted 1:5 and injected on the liquid chromatography system nLC Ekspert nanoLC400 set in nanoconfiguration coupled with Triple TOF 6600. Chromatography was performed using an in-house packed nanocolumn Kinetex EVO C18, 1.7 μm, 100 A (Phenomenex, Torrance, CA, USA), 0.75 × 100 mm at room temperature. The gradient started at 5% mobile phase B and was linearly increased to 100% B in 5 min, maintained for 45 min, then returned to the initial ratio in 2 min, and maintained for 8 min at a flow rate of 150 nl/min. The samples were analyzed in technical duplicate, in positive mode with electrospray ionization. Data acquisition and processing were performed with Analyst TF (version 1.7.1, AB SCIEX, Foster City, CA, USA). The following parameters were used: CUR 10 psi, GAS1 0 psi, GAS2 0 psi, source temperature 80 °C, capillary voltage 2000 V. Spectra were acquired by full-mass scan from 200 to 1700 m/z and information-dependent acquisition (IDA) from 50 to 1800 m/z (top 8 spectra per cycle). The declustering potential was fixed at 80 eV, and the collision energy was fixed at 40 eV; target ions were excluded for 20 s after two occurrences. Wiff files were processed using the open-source software MS-DIAL version 4.60. The parameters were as follows: MS1 tolerance = 0.01 Da, MS/MS tolerance = 0.025 Da, MS1 mass range = 100 to 1700 Da, and minimum peak height = 10,000 amplitude. All other parameters were kept at the default values. Lipids were annotated using the MS-DIAL internal lipid database. Only the lipids showing MS/MS spectral similarity to the reference spectra in the MS-DIAL internal database. The peak areas of annotated lipids were normalized by sum and statistically analyzed using the freely available online software metaboanalyst (https://www.metaboanalyst.ca/), or were Zscore-transformed and statistically analyzed using Perseus software. Lipid ontology analysis of significant lipids was performed with The Lipid Ontology (LION) software (http://www.lipidontology.com/). Targeted analysis of FA in positive mode Wiff files were processed by using LipidView software as done previously [30]. The software enables identification and characterization through database searching of MS/MS fragment ions profiling various lipid classes by lipid head groups, fatty acid and long chain base characteristic fragments and neutral losses both in positive and in negative mode.

### Free fatty acids consumption /release assay

50.000 cells were seeded in 24 multi-well plates in complete medium. The day after, the culture medium was replaced with medium with DMSO or 3I 5 μm for different time points as indicated in the graph. A blank control with complete medium was used to measure the metabolite relative quantification and consumption. The day of the extraction cells were counted from each plate to calculate the rate of metabolite uptake / secretion per cell. Negative values corresponded to metabolites up-taken from the medium, whereas positive values corresponded to metabolites released in the medium. For MS analysis of the extracellular medium, 400 µl of cell culture medium were collected and metabolite extraction was performed [31]. Briefly, collected medium was centrifuged 5 min at 4 °C at maximum speed to eliminate dead cells and debris. 80uL of the supernatant was added to 400 µL of metabolomics extraction buffer ( 40% LC-MS grade ACN, 40% LC-MS Methanol, 20% ultrapure water with 10 μm of Valine d8 [CK isotopes, DLM-488] as internal standard) (on ice), vortexed and incubated for 15 min in a Thermomixer (4 °C) maximum speed. After incubation with the metabolomics extraction buffer cells were centrifuge at max speed for 20 min. Then supernatants were collected into autosampler vials for UHPLC-MS analysis. The cell number was counted to normalize lipid uptake / secretion per cell. Metabolites were identified and quantified using a UHPLC Vanquish Flex coupled to a Exploris 240 mass spectrometer (Thermo Fisher, Bremen, Germany). Five microliters of sample were injected onto a Sequant ZIC-pHILIC column (150 × 2.1 mm, 5 μm) and guard column (20 × 2.1 mm, 5 μm) (both Merck Millipore) for the chromatographic separation, as previously described by MacKay GM,2015 [31] with few modifications. Briefly, the mobile phase was composed of 20 mM ammonium carbonate and 0.2% ammonium hydroxide in water (mobile phase A), and acetonitrile (mobile phase B). The flow rate was set at 0.2 mL/min with the following gradient: 80% B for 2 min, linear decrease to 30% of B 15 min and then solvents were brought back to starting conditions and kept for 6 min. The column oven temperature was maintained at 40 °C for the entire run. Mass spectrometer was operated in Full MS and ddMS2 (switching polarity mode) with the following parameters: mass range of 70–900 m/z with polarity switching mode, 60,000 of resolution for full MS and 30,000 resolution for the ddMS2, normalized collision energies were set to 20, 40, 80 values, AGC target and maximum injection time were set respectively to standard and auto for both full MS and ddMS2 scan. Metabolites were identified using acquired fragmentation spectra matched with online and local libraries. Metabolites were then quantified using Xcalibur Quan Browser software and Compound discoverer 3.3 (Thermo Fisher). Metabolites intensities were normalized by either total ion count (TIC) or internal standard intensities.

### Lipid mix Titration

To evaluate cell growth dependency on lipids availability, cells were grown in delipidated complete medium (dFBS, Fetal Bovine Serum (FBS) South America, Lipid Depleted, BIOWEST, S181L) with lipid supplements (Chemically Defined Lipid Concentrate from Gibco, 11905031). Cells were seeded at the same cell density described above and cultured for 2 days, in complete medium supplemented with different amount of lipid concentrate a described in the manuscript. After incubation, cells were counted and lysed as described before.

## Results

### Enhanced lipid metabolism and lipid droplet storage correlate with BACE2 expression in melanoma and pancreatic cancer

**Fig. 1:**
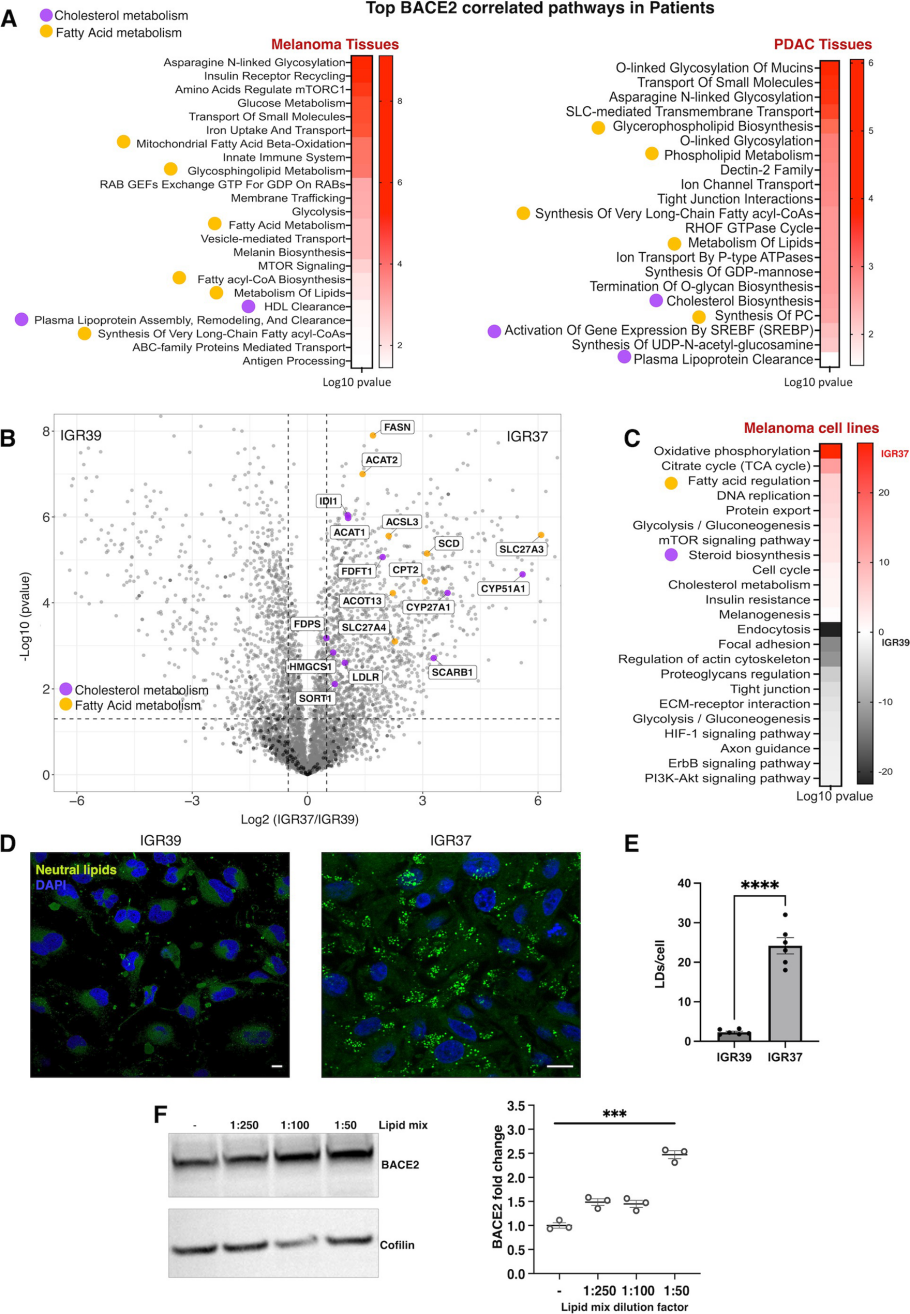
BACE2 expression is associated with lipid metabolism and lipid droplets accumulation in cancer. (**A**). KEGG pathway analysis of TOP 500 genes co-expressed with BACE2 (R^2^ > 0.40) in melanoma and PDAC tumor biopsies explored by GEPIA. Pathway category and relative enrichment score (Log *p* value) were plotted as heatmap. **(B)**. Volcano plot showing proteomics data of IGR37 vs. IGR39. Colored points indicate proteins belonging to pathways, highlighted in the legend, that display high fold-changes (x axis) and high statistical significance (-Log10 of *p* values, y axis) in IGR37 compared to IGR39. Dashed horizontal line shows the *p* values cut off FDR < 0.05, and the two vertical dashed lines indicate down/up regulated proteins (|Log2 fold-change| >1). **(C)**. Functional annotation of proteins significantly upregulated in IGR37 vs. IGR39. KEGG pathway enrichment analysis was performed by EnrichR. Significant enriched pathway categories (*p* value < 0.05) with their relative enrichment score (−/+ Log *p* value in IGR37/IGR39, respectively) were plotted as heatmap. **(D)**. Visualization of lipid droplets (LDs) with Nile Red staining. A representative fluorescence confocal microscopy image of melanoma cells is shown using a double staining with DAPI (blue), as nuclear marker, and Nile Red (green), as neutral lipids dye. **(E)**. Quantification of LDs per cell by immunofluorescence analysis using Fiji software. T-test statistical analysis of LDs per cells was applied for the cell lines reported in the figure. (T-test ****p value < 0.0001. *N* = 6). Scale bar: 10 μm. **(F)**. Western blot of BACE2 expression and boxplot of its fold change in IGR37 cells treated with different amount of lipid mix added to delipidated serum

BACE2 has been implicated in cancer development and progression of melanoma and pancreatic adenocarcinoma (PDAC), where its activity has been shown to promote cancer cell proliferation [20, 21]. However, the mechanisms through which BACE2 contributes to tumor growth remain unclear. To address this issue, we investigated genes co-expressed with BACE2 in melanoma and PDAC patient tissues using the Gene Expression Profiling Interactive Analysis (GEPIA) tool, which integrates TCGA and GTEx gene expression datasets. By pathway enrichment analysis of top 500 similarity genes, we found that BACE2 expression level correlates with genes involved in fatty acid and cholesterol metabolism (Fig. 1A), suggesting a link between BACE2 expression and lipid metabolic regulation in cancer.

To investigate these findings at cellular level, we focused on cancer cell lines with the highest BACE2 expression. Among various tumor types in the Cancer Cell Line Encyclopedia (CCLE), melanoma exhibited the highest BACE2 levels across 1479 profiled cell lines (Fig. S1A). We performed comprehensive multi-omics characterization of melanoma cell lines IGR37 and WM266.4, both expressing higher BACE2 levels compared to their isogenic counterparts IGR39 and WM115, respectively (Fig. S1B). Global proteomic analysis of the IGR cell lines identified and quantified 5247 protein groups, with statistical testing revealing 3,124 proteins significantly regulated in IGR37 compared to IGR39 (Tables S1-2, Fig. 1B). Consistent with data from patients tissues, IGR37 cells showed increased expression of proteins involved in lipid regulation (Fig. 1C). These included enzymes critical for fatty acid and cholesterol biosynthesis such as ACSL3, FASN, ACACA, FDPS, CYP51A1, HMGCS1, and FDFT1. Similarly, proteomic analysis of WM266.4 versus WM115 cells showed upregulation of lipid metabolic proteins (Fig. S1C, Tables S3-S4). These findings support the hypothesis that BACE2 plays a significant role in regulating lipid metabolism in cancer cells.

To determine whether these proteomic changes were reflected at the lipid level, we performed untargeted lipidomics analysis [30]. Out of 473 lipid species detected, 283 were significantly altered between IGR37 and IGR39 cell lines (Tables S5-6). In particular, IGR37 cells displayed higher level of sphingomyelins (SM), sterols (ST) and triacylglycerols (TAGs) (Fig. S1D). Lipid ontology (LION) enrichment analysis revealed overrepresentation of TAG metabolism pathways, increased lipid storage, and LDs formation in IGR37 cells (Fig. S1E). Since LDs are composed primarily of a neutral lipid core, formed by TAGs and sterol esters [32], we used confocal microscopy with Nile Red staining to visulalize LDs in melanoma cells [33]. As predicted by the lipidomics data, cells with elevated BACE2 expression displayed marked accumulation of LDs (Fig. 1D, E, Fig. S1F). Overall, these results suggest that BACE2 expression is associated with enhanced lipid storage in cancer cells.

Given this strong correlation between BACE2 expression and lipid accumulation, we hypothesized that cancer cells may modulate BACE2 protein level in response to extracellular lipid availability. Indeed, when we exposed IGR37 cells to increasing concentrations of a chemically defined lipid mix, we observed a dose-dependent increase in both BACE2 protein abundance (Fig. 1F) and cell proliferation (Fig. S1G). These results suggest a reciprocal relationship in which lipid availability regulates BACE2 levels, which in turn may function as a sensor and regulator of lipid utilization, ultimately influencing cancer cell proliferation.

### Fatty acids and cholesterol transporters are shedded by BACE2 into the extracellular space

**Fig. 2:**
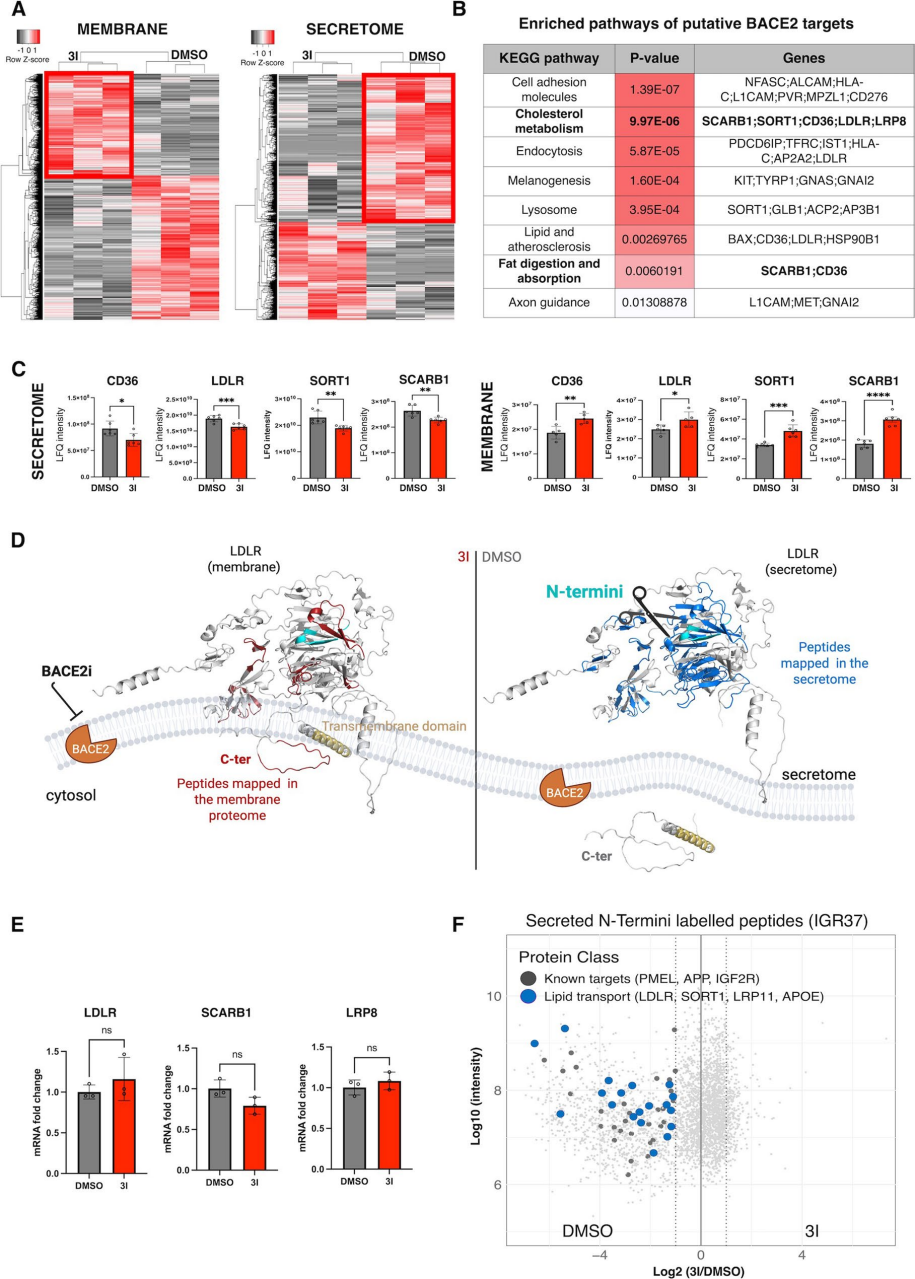
Identification of BACE2 targets via spatial proteomics and N-Terminomics.** (A)**. Spatial proteomics analysis of proteins affected by BACE2 inhibition. Hierarchical clustering analyses (Heatmap) of all differentially expressed proteins in membrane proteome and secretome analysis, in IGR37 treated with 3I and DMSO as control (Student T-test FDR < 0.05. *N* = 3 biological replicates in technical duplicates). Red, high expression; grey, low expression. (**B**). Functional annotation of the 60 BACE2 putative targets (proteins that are statistically less abundant in the secretome of cells treated with DMSO and that concomitantly accumulate in the membrane proteome in 3I treated cells). KEGG pathway enrichment analysis performed by using the web tool EnrichR (*p* value < 0.05) is represented. **(C)**. Boxplot of Z-score intensity values from secretome (left panel) and membrane proteome (right panel) analysis of several lipid transporters assigned as BACE2 targets. **(D)**. Cartoon, made with BioRender (https://biorender.com/), showing BACE2 shedding activity on LDLR transporter. Peptides mapped by mass-spectrometry analysis in membrane proteome when BACE2 is inactive (left side, red structures) and in the secretome when BACE2 is active (blue structures) are highleted on the *AlphaFold* predicted 3D structure of LDLR. Sequence cyan colored residing in the soluble part of the protein corresponds to the newly generated N-termini when BACE2 is active. **(E)**. Lipid transporters genes mRNA fold change in IGR37 cells treated with DMSO or 3I for 48 h. (Student T-test ns = not significant). *N* = 3. **(F).** Scatter plot of DMSO (active protease) /3I ratio labelled peptides from N-TAILS analysis of IGR37 cells. As protease cleavage generates new N-termini, the DMSO (active protease) /3I ratio (≥ 2.0 or heavy singleton) corresponds to protease-generated neo-N-termini. These are distinguished from background proteolysis products and original mature N-terminal peptides that have an isotope ratio of 1:1, as they are equally abundant in both samples. Among the neo generated N-termini, known BACE2 targets are labeled in dark gray while lipid transporters are labeled in blue

To investigate the molecular link between BACE2 activity and lipid metabolism, we searched for unknown BACE2 targets involved in this process. Specifically, we examined spatial proteomics and secretomics changes in BACE2-inhibited cells to detect shedded transmembrane targets. For this purpose, we employed a selective BACE2 inhibitor (BACE2i), referred to as 3I. As both BACE2 and its targets are transmembrane proteins, once in proximity, BACE2 cleaves its substrates releasing their ectodomain into the extracellular space [34]. In contrast, BACE2 inhibition prevents this cleavage, resulting in the accumulation of full-length substrates at the plasma membrane. We thus analyzed proteins that accumulated in the membrane and were concomitantly decreased in the secretome following BACE2 inhibition (Fig. 2A, Tables S7-10). Membrane proteins were isolated using a commercial dedicated extraction protocol, and the fractionation quality was confirmed by Western Blot using anti-E-cadherin as marker of membrane fraction, and anti-Histone H3 and PMEL as markers of the nuclear and cytosolic fractions respectively (Fig. S2A). Gene Ontology analysis confirmed the enrichment of membrane-associated proteins (Fig. S2B), while pathways enrichment analysis of the proteins significantly regulated upon BACE2i revealed an alteration of membrane vesicles transport, in particular of receptor mediated endocytosis and lipid transport (Fig. S2C). By intersecting proteins enriched in the membrane fraction upon BACE2i and proteins more abundant in the secretome of BACE2 proficient cells, we identified 60 putative BACE2 targets. In addition to known targets involved in melanogenesis, we detected proteins involved in cell adhesion and in lipid transport, such as CD36, LDLR, SORT1 and SCARB1 (Fig. 2B, C). Seven of the 60 targets have been previously reported as BACE2 potential targets in pancreatic β-cells (Fig. S2D) [35], while BACE2 mediated LDLR cleavage was recently decribed in liver cancer [22]. Importantly, BACE2-mediated cleavage of LDLR was evident also by peptide mapping. In BACE2-active conditions, secretome peptides mapped exclusively to the LDLR extracellular domain, whereas BACE2 inhibition resulted in membrane accumulation of both extracellular and intracellular domains, supporting the evidence of a proteolytic cleavage (Fig. 2D).

Additionally, mRNA levels of lipid transporters LDLR, SCARB1, and LRP8 remained unchanged following BACE2 inhibition (Fig. 2E), indicating that the increased abundance of these transporters on the plasma membrane derives from post-translational regulation, via reduced shedding, rather than from enhaced transcription.

To validate potential targets, we employed N-TAILS degradomics (N-TAILS) [36–38] that enables the proteome-wide identification, mapping and quantification of protein N-termini. N-TAILS leads to protease substrates profiling by labeling and enriching native and neo-N-terminal peptides, generated by proteasic activity. We applied N-TAILS to analyze the secretome of melanoma cells with or without BACE2 inhibition. Primary amines were differentially labelled by reductive dimethylation in the compared conditions and N-terminal peptides were negatively selected through polymers that seize free amines generated by tryptic digestion [36–38]. By mass spectrometry, we identified a total of 3618 N-termini modified by dimethylation and 447 endogenously acetylated (Fig. S2E, Tables S11-12). We searched for non-canonical N-termini enriched in untreated cells, as potential BACE2 cleavage sites. Together with BACE2 known targets as PMEL and APP, we found neo N-termini in proteins involved in lipid transport such as LDLR, SORT1 and LRP1 (Fig. 2F, Table S11), strongly supporting their direct cleavage by BACE2. To confirm the robustness of these findings, we replicated the N-TAILS analysis in another melanoma cell line, WM266.4, identifying 225 conserved substrates, including LDLR (Fig. S2E-F, Tables S11-12). Collectively, the integration of spatial proteomics and N-TAILS data provides compelling evidence that BACE2 regulates the shedding of membrane-associated lipid transporters into the extracellular space.

To validate the specificity of these BACE2-regulated substrates and rule out drugs’ off-target effects, we performed genetic manipulation of BACE2 expression. Specifically, we overexpressed BACE2 in HEK293T cells (Fig. S2G), a cell line with intrinsically low BACE2 expression, as reported in the Human Protein Atlas (https://www.proteinatlas.org/ENSG00000182240-BACE2/cell+line). We then analyzed the secretome of BACE2 overexpressing HEK293T cells to assess whether candidate substrates were shed in a BACE2-dependent manner. Secretome profiling revealed a significant enrichment of lipid transporters, such as LDLR, SORT1, and LRP1, as well as established BACE2 targets including PMEL and IGF2R in BACE2-overexpressing cells (Fig. S2H, Tables S13–S14).

### CD63 facilitates BACE2-dependent shedding of its substrates into the extracellular space

**Fig. 3:**
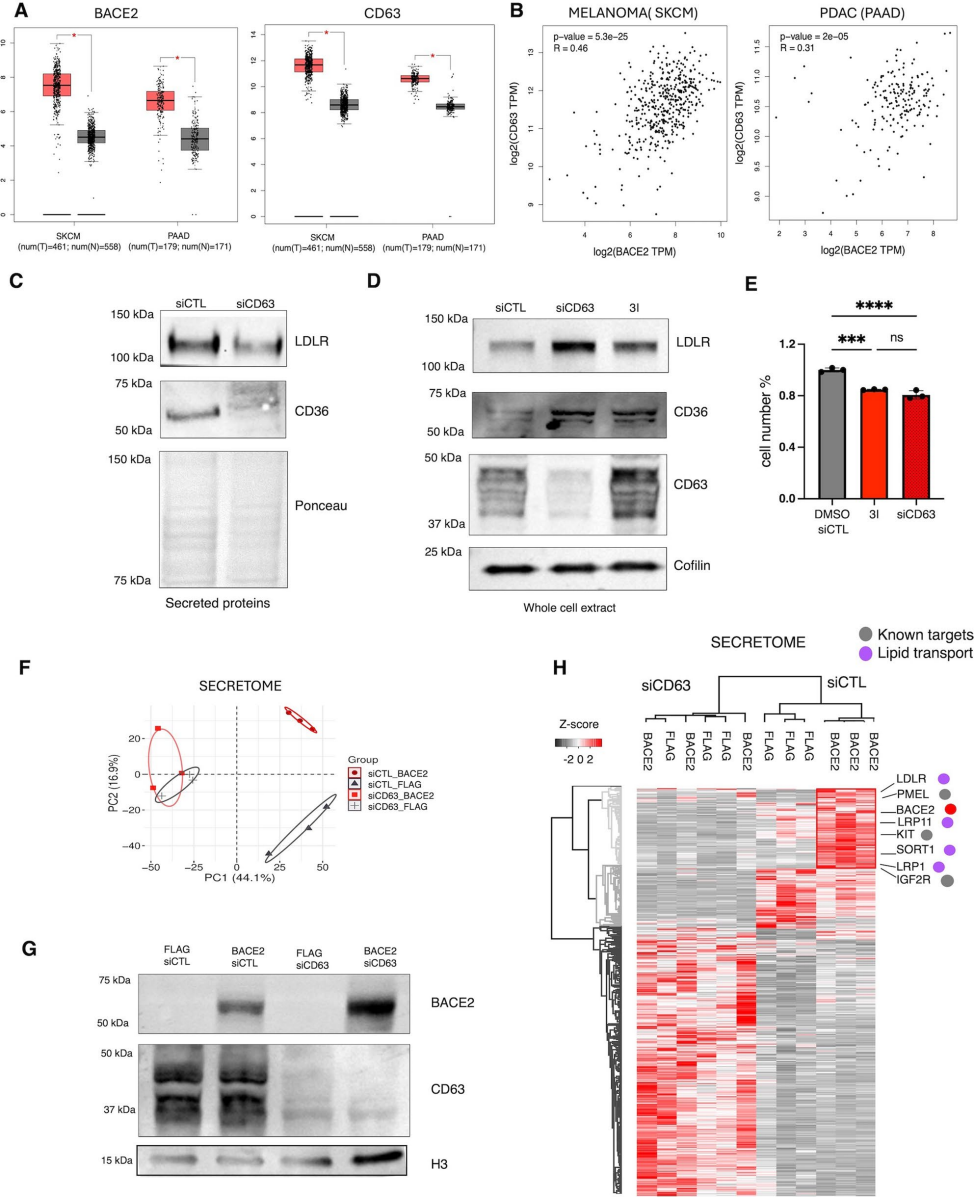
CD63 facilitates BACE2-dependent shedding of lipid transporters. (**A**). BACE2 and CD63 expression profiling in melanoma and PDAC explored by GEPIA. The gene expression profile across tumor samples (red boxes) and paired normal tissues (grey boxes) is reported. Each dot represents the level of expression/ sample. (**B**) Gene correlation analysis between BACE2 and CD63 explored by GEPIA in melanoma and PDAC. Each dot represents the level of expression (Transcript Per Million TPM) for CD63 and BACE2. P-value of correlation analysis was also reported fro each cancer type. (**C**) Western blot of lipid transporters LDLR and CD36 secreted in the medium by IGR37 cells upon 48 h of CD63 silencing (siCTL vs. siCD63). Ponceau was used as loading control. (**D**) Western blot of CD63 and lipid transporters (LDLR and CD36) in whole cells extract of IGR37 cells upon 48 h of CD63 silencing (siCTL vs. siCD63) and BACE2 inhibition (3I). Histone H3 was used as loading control. (**E**) Analysis of cell viability for IGR37 cells upon 48 h of CD63 silencing (siCTL vs. siCD63) and of BACE2 inhibition (3I). (Student T-test **** *p* value < 0.0001,***p value < 0.001). (**F**) Principal Component Analysis (PCA) of secreted proteins measured by quantitative LC-MS/MS analyses of HEK293T cells transfected with BACE2-Flag or Flag empty vector as control plus CD63 silencing (siCTL vs. siCD63) as indicated in the plot. *N* = 3 biological replicates. (**G**) Western blot of BACE2 and CD63 in HEK293T cells transfected with BACE2-Flag or Flag empty construct as control plus CD63 silencing (siCTL vs. siCD63) as indicated in the plot. *N* = 3 biological replicates. Histone H3 was used as loading control. *N* = 3. (**H**) Secretome analysis of HEK293T cells transfected with BACE2-Flag or Flag empty vector as control plus CD63 silencing (siCTL vs. siCD63) as indicated in the plot. Hierarchical clustering analyses (Heatmap) using unsupervised Euclidean distance of all differentially expressed proteins in the secretome analysis (Anova test *p* value < 0.05. *N* = 3 biological replicates). Red, high expression; grey, low expression

Given that BACE2-mediated cleavage requires substrate proximity within specific membrane microdomains, as previously described for PMEL maturation [39], we investigated whether similar molecular scaffolds could facilitate BACE2 interaction with lipid transporters. Among BACE2-associated tetraspanins, CD63 emerged as a strong candidate, being highly expressed in melanoma and PDAC cells (Fig. 3A), showing strong correlation with BACE2 levels (Fig. 3B) and known to organize BACE2 driven PMEL maturation in melanosomes [39]. It has been demonstrated that CD63 directly assists in sorting the luminal domain of PMEL into intraluminal vesicles, where BACE2 cleavage enables amyloid fibril assembly during melanosome maturation.

To understand if CD63 is also involved in BACE2-mediated shedding of lipid trasporters, we silenced CD63 in IGR37 melanoma cells (Fig. 3C, D) and, by western blot analysis, we observed a reduction of the shedding of BACE2 substrates as LDLR and CD36 (Fig. 3C). These data show that CD63 silencing phenocopies BACE2-inhibited cells, displaying similar intracellular lipid transporters accumulation and reduced cellular proliferation (Fig. 3D, E). Moreover, BACE2 overexpressed in HEK293 cells fails to shed its substrates when CD63 is absent further supporting a functional link between BACE2 and CD63, Indeed, CD63 knockdown impaired the secretome changes normally induced by BACE2 overexpression, as shown by the PCA plot (Fig. 3F, G), suggesting that CD63 loss compromises BACE2 activity. Specifically, CD63 depletion impaired BACE2-dependent shedding of all its substrates, including lipid transporters (Fig. 3H, Tables S15-S16). Collectively, these data demonstrate that CD63 organizes BACE2 activity, providing a mechanistic basis for lipid transporter cleavage analogous to that observed in PMEL maturation.

### BACE2 Inhibition reprograms lipid metabolism in cancer and normal cells

**Fig. 4:**
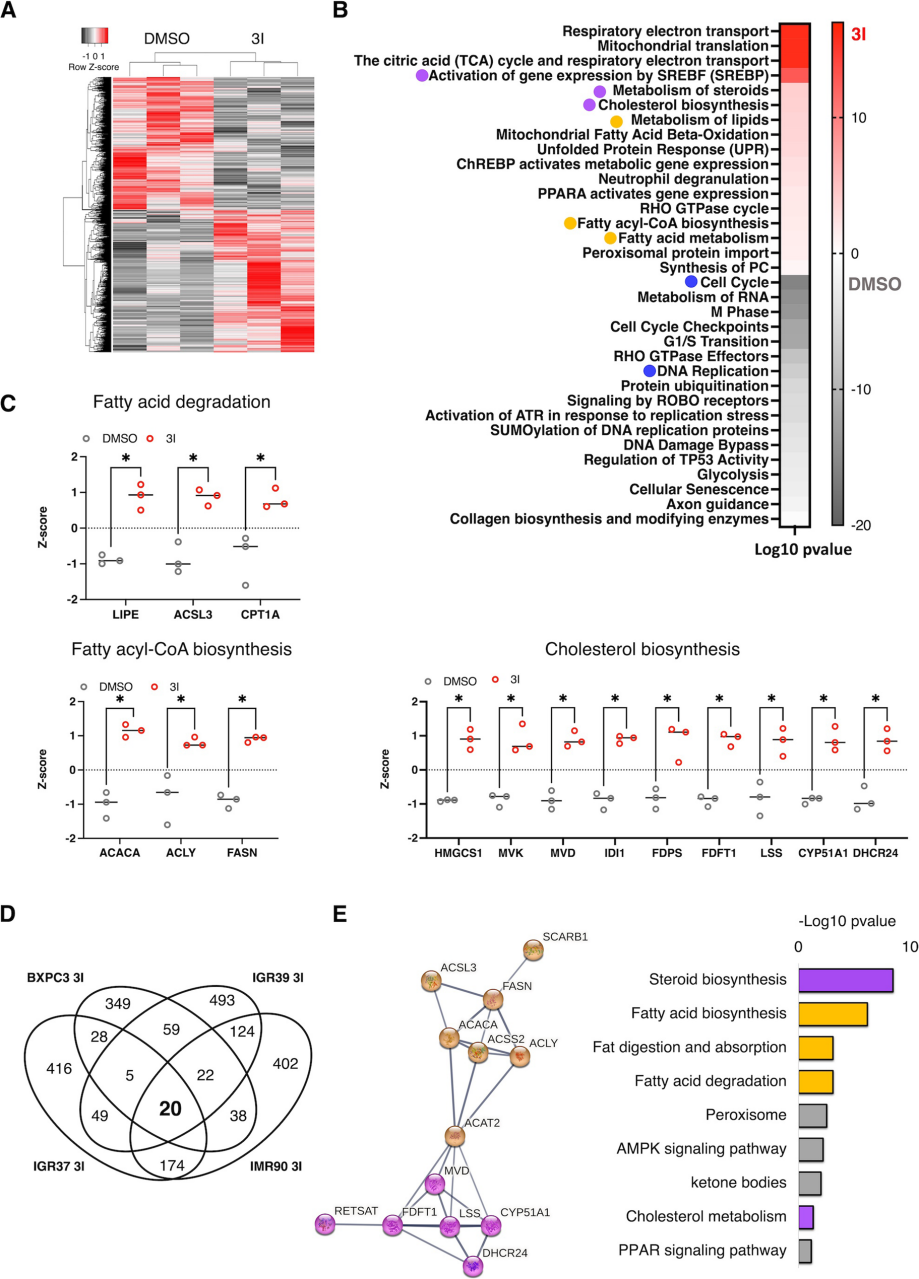
BACE2 modulates lipid metabolism.** (A)**. Label-free proteome quantification of IGR37 treated with 3I 5 µM or DMSO as control for 48 h. Hierarchical clustering analysis (Heatmap) using unsupervised Euclidean distance of differentially expressed proteins between IGR37 treated with 3I or DMSO (Student T-test permutation-based FDR < 0.05. *N* = 3 biological replicates in technical duplicates. Each row in the figure represents a protein, and each column represents a sample. Red, high expression; grey, low expression. **(B).** Functional annotation of proteins significantly upregulated in IGR37 treated with 3I and DMSO. KEGG pathway enrichment analysis performed by the web tool EnrichR. Pathway category and their relative enrichment scores (−/+ Log *p* value in 3I (red) or DMSO (grey) were plotted as heatmap. **(C)**. Selected proteins upregulated in BACE2 inhibited cells (as indicated in the dotplots of Z-score values from proteomic analysis) and their biological role in lipid metabolism. **(D)**. Venn diagram of proteins significantly upregulated upon 3I treatment in melanoma, PDAC cells and fibroblasts. **(E)**. Functional protein association networks by using STRING vs12.0 (https://string-db.org/) of the shared proteins from the Venn diagram on the left and related KEGG pathway enrichment analysis

To investigate how BACE2-dependent shedding of lipid transporters impacts intracellular metabolism, we performed a global proteomic analysis of IGR37 following BACE2 inhibition. Among the 6638 quantified proteins (Tables S15,16), 1432 were significantly altered (Fig. 4A). Coherently with our previous studies [20, 21], where we observed that BACE2 inhibition reduced cell proliferation, pathway enrichment analysis revealed a downregulation of proteins related to cell cycle and DNA replication (Fig. 4B, blu dots and Tables S17,18). In contrast, proteins related to lipid metabolism were markedly upregulated (Fig. 4B, yellow and violet dots). Notably, we observed increased expression of key lipolytic enzymes such as LIPE, along with ACSL3 and CPT1, which are essential for fatty acid activation and mitochondrial transport. Enzymes driving de novo lipogenesis, as FASN, ACACA, and ACLY, and those involved in cholesterol biosynthesis were also upregulated (Fig. 4C), indicating an overall enhancement of lipid turnover.

These changes were consistently observed across other models, including WM266.4 metastatic melanoma cells (Fig. S3A, Tables S19–20) and two pancreatic cancer cell lines, BXPC3 and CAPAN2 (Fig. S3B–C, Tables S21–24). Similarly, in IGR39 cells, which have lower BACE2 expression, BACE2 inhibition still triggered upregulation of lipid metabolic proteins (Fig. S3D, Tables S25–26). These findings indicate that BACE2 modulates lipid metabolism across diverse cancer contexts.

To assess whether this regulatory role extends beyond cancer, we queried the DepMap portal and found that BACE2 mRNA expression is also elevated in fibroblasts, at levels comparable to cancer cell lines (Fig. S3E–F). We confirmed BACE2 expression in IMR90 lung fibroblasts (Fig. S3G), and, upon BACE2 inhibition, observed upregulation of proteins involved in cholesterol and FA metabolism, mirroring changes seen in cancer cells (Fig. S3H, Tables S27–28). By intersecting the proteomics data from all BACE2-inhibited cell lines, melanoma, PDAC, and fibroblasts, we identified 20 commonly upregulated proteins. These include enzymes linked to cholesterol biosynthesis and fatty acid metabolism (Fig. 4D–E). To exclude off target effects of the inhibitor 3I, we validated these findings by BACE2 knockdown in IGR37 cells (Fig. S3I), confirming enrichment of the same lipid-associated proteins (Fig. S3J, Tables S29–30). Collectively, these results demonstrate that BACE2 activity modulates core lipid metabolic pathways in both cancerous and non-cancerous cells.

### BACE2 mediated shedding of lipid transporters controls lipid uptake and LDs dynamics

**Fig. 5:**
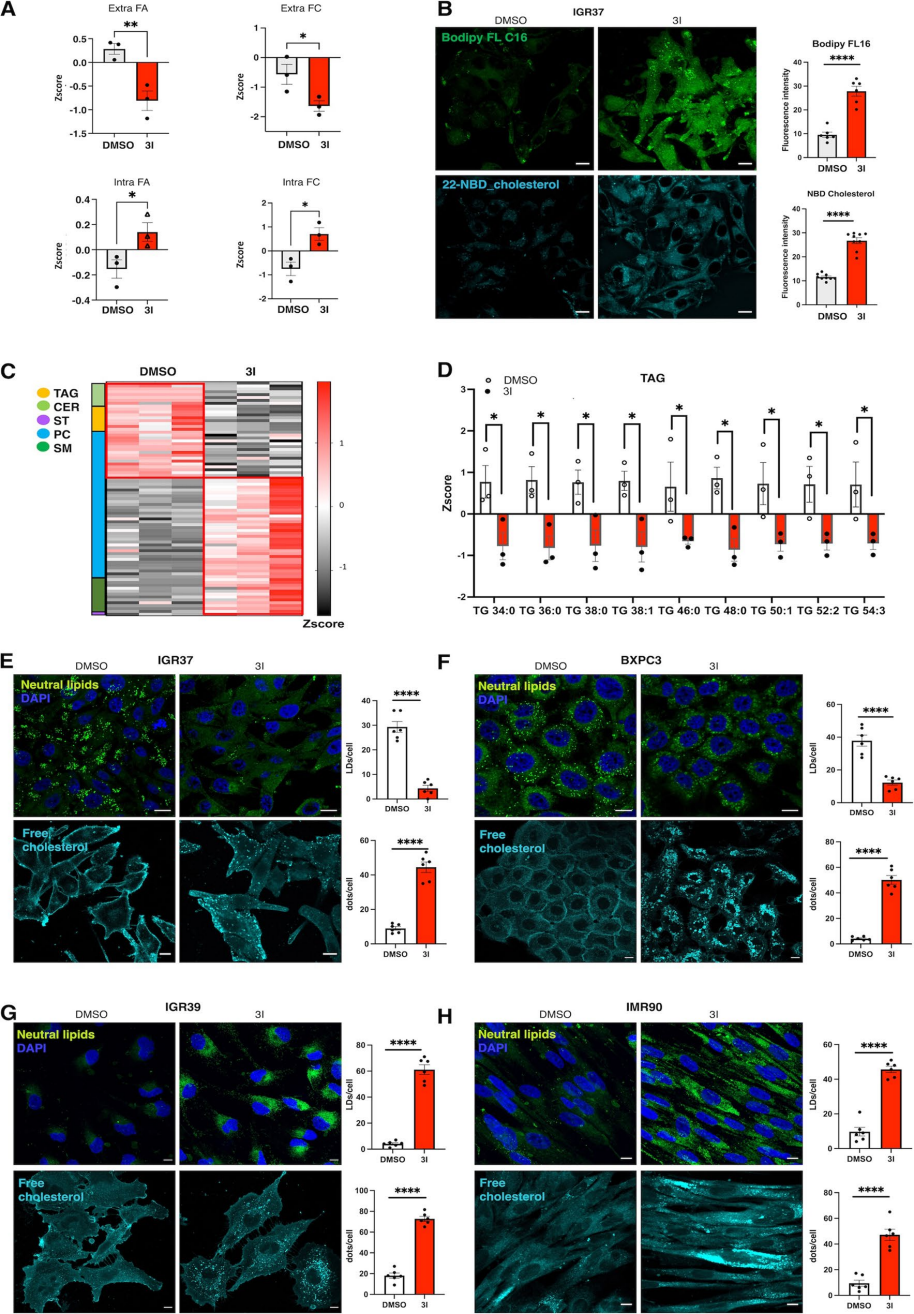
Analysis of lipid uptake after BACE2 inhibition and its effect on LDs and free cholesterol distribution.** (A)**. Analysis of free Fatty Acids (FA) grouped in one class and Free Cholesterol (FC) uptake and intracellular accumulation in IGR37 cells treated with DMSO and 3I for 48 h. Upper panels. Histograms of Zscore averaged values of FA and FC from extra-lipidomics analysis. Lower panels. Histograms of Zscore averaged values of FA and FC from intra-lipidomics analysis. *N* = 3 in technical duplicates. **(B)**. Upper panel. BODIPY-C16 tracks fatty acid uptake in IGR37 cells. Cells were treated for 1 h with BODIPY-C16 5µM and analyzed by confocal microscopy, bar = 10 μm. Histogram of mean fluorescence intensity is reported in the graph on the right (Student T-test **** *p* value < 0.0001). Lower panel. 22-NBD-Cholesterol tracks FC uptake in IGR37 cells. Cells were treated for 1 h with NBD-cholesterol 4µM and analyzed by confocal microscopy, bar = 10 μm. *N* = 7. Histograms of mean fluorescence intensity is reported in the graph on the right (Student T-test **** *p* value < 0.0001). (**C**). Heatmap analysis of significant lipid species in IGR37 treated with DMSO or 3I 5 µM for 48 h. Each colored cell on the map corresponds to a Z-score value. Each raw corresponds to a lipid species. Each lipid specie is grouped in lipid classes as reported in the graph (T-test *p* value < 0.05; *N* = 3 biological replicates in technical duplicates). **(D).** Boxplot of Z-score values of TAG species measured by lipidomics analysis of IGR37 DMSO vs. 3I, (T-test analysis: *p value < 0.05). **(E-H)**. Visualization of lipid droplets (LDs) with Nile Red staining and of endogenous free cholesterol with Filipin staining in DMSO vs. 3I treated cells. A representative fluorescence confocal microscopy image of melanoma, PDAC and fibroblasts cells is shown using DAPI (blue) as nuclear marker, Nile Red (green) as LDs marker, or Filipin (cyan) as free cholesterol marker. Scale bar: 10 μm

Among BACE2 targets, we identified two key lipid transporters: LDLR, essential to maintain cholesterol homeostasis [40], and CD36, a master regulator of fatty acid uptake [41]. We then hypothesized that BACE2 regulates lipid metabolism by modulating the shedding of these transporters and, in turn, influencing lipid uptake. To address this question, we measured the level of cholesterol and fatty acids both intra- and extra-cellulary through lipidomics analysis in IGR37 cells. As expected, we observed an increased consumption of both FAs and cholesterol in BACE2 inhibited cells compared to untreated cells, mirroring their intracellular accumulation (Fig. 5A, Tables S34-35). Immunofluorescence-based uptake assays using Bodipy FL C16 (a fluorescent palmitate analogue) and 22-NBD-cholesterol (a fluorescent cholesterol analogue) confirmed enhanced lipid uptake (Fig. 5B). Importantly, Bodipy FL C16 uptake relies exclusively on active transport by lipid transporters and not on passive diffusion, highlighting the role of transporter-mediated uptake [42]. Similar results were observed in BXPC3, IGR39 and IMR90 cells (Fig. S4A). Moreover, increased lipid uptake was also observed in IGR37 upon CD63 silencing, further attesting CD63 role in sustaining BACE2 mediated lipid transporters shedding (Fig. S4B).

To understand how enhanced lipid uptake reflects on intracellular lipid state, we performed mass spectrometry based global lipidomics analysis of IGR37 cells treated with 3I. As observed for free cholesterol, this analysis revealed a marked accumulation of phospholipids, sphingomyelins, alongside a reduction in TAGs and ceramides (Fig. 5C, Tables S34,35). These results are consistent with the lipid metabolic rewiring predicted by proteomics analysis.

Coherent with the decrease in TAGs content (Fig. 5D) and intracellular cholesterol accumulation (Fig. 5A), confocal imaging showed that BACE2 inhibition caused a reduction in LDs and a formation of intracellular cholesterol deposits in IGR37 and BXPC3 cells (Fig. 4E, F). Differently, in IGR39 cells and IMR90 fibroblasts, excess of lipids were sequestred in newly formed LDs (Fig. 5G, H). These divergent responses reflect the different baseline lipid storage states of high- versus low-BACE2 cells, as shown in Fig. 1. Time-course imaging revealed that LDs breakdown and accumulation begin within 12 h of BACE2 inhibition and persist for at least 48 h (Fig. S4C, D; Appendix movies 1–2), suggesting that LDs remodeling is an early and direct consequence of BACE2 activity loss.

In summary, these findings demonstrate that BACE2 governs lipid uptake by controlling the availability of lipid transporters at the plasma membrane, thereby influencing intracellular lipid levels and LDs homeostasis.

### Enhanced lipid uptake activates PPARα signaling via FA accumulation

**Fig. 6:**
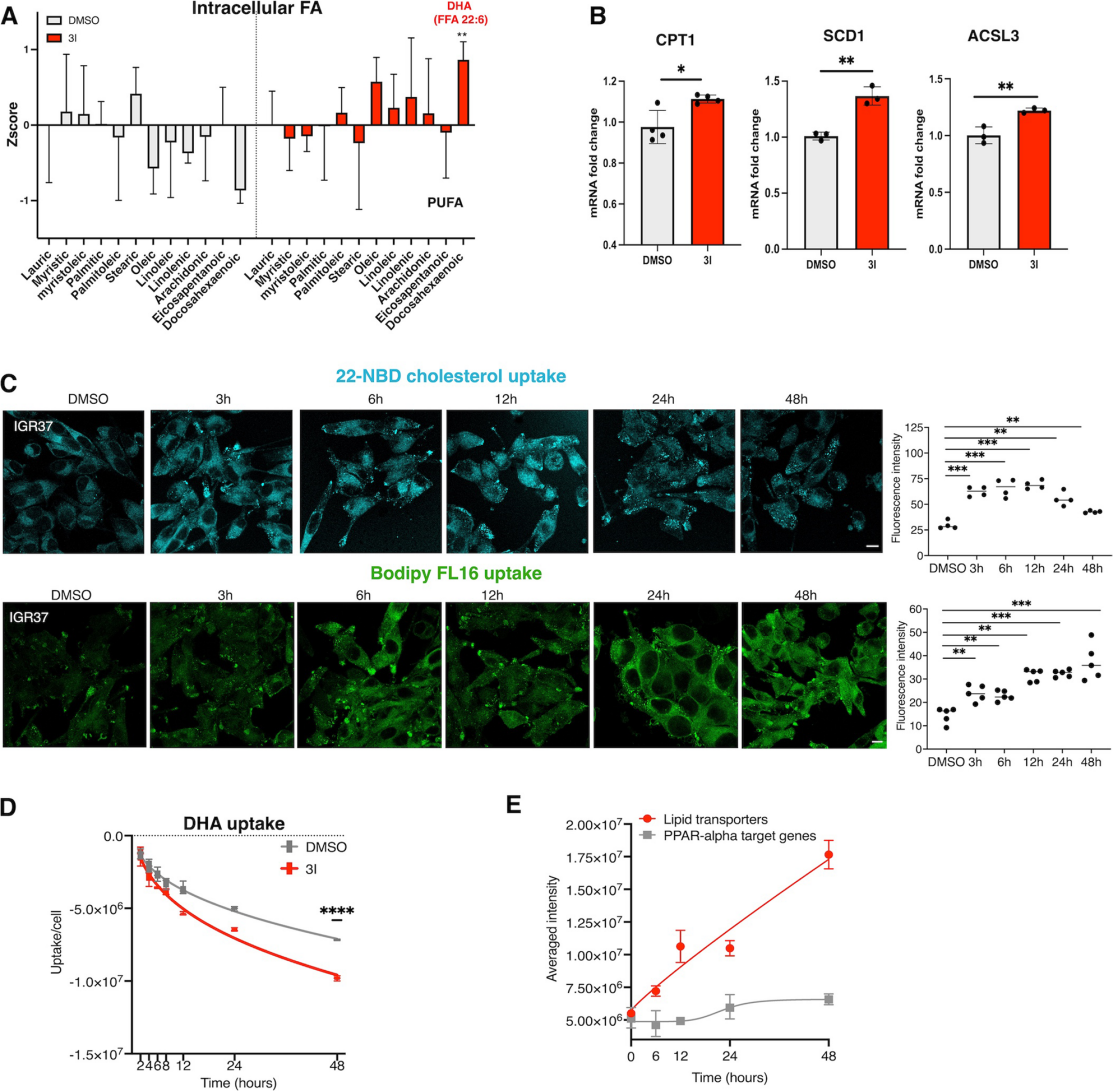
BACE2 inhibition impact on PPAR-alpha activation.** (A)**. Histograms of Zscore values for each FA specie from intra-lipidomics analysis of IGR37 cells treated with DMSO and 3I for 48 h. (Student T-test ****p value < 0.0001). **(B)**. PPAR-alpha target genes, CPT1, SCD1, ACSL3, mRNA fold change in IGR37 cells treated with DMSO and 3I for 48 h (Student T-test **p value < 0.01, *p value < 0.05). *N* = 3. **(C)**. Time course analysis of lipid uptake upon BACE2 inhibition. Upper panels: cells were treated for 1 h with BODIPY-C16 5µM and analyzed by confocal microscopy, bar = 10 μm. Values of mean fluorescence intensity are reported in the graph on the right (Student T-test ***p value < 0.001, **p value < 0.01). *N* > 6. Lower panels: cells were treated for 1 h with NBD-cholesterol 4µM and analyzed by confocal microscopy, bar = 10 μm. *N* = 7. Histograms of mean fluorescence intensity was reported in the graph on the right (Student T-test ***p value < 0.001, **p value < 0.01). **(D).** Kinetics of DHA consumption rate in the media upon 3I treatment. IGR37 cells were treated with 5µM 3I till 48 h as showed in the graph. DHA uptake was measure by LC-MS/MS. MS signals were normalized by the number of cells. *N* = 5. (Student T-test ***p value < 0.0001). **(E).** Membrane proteomics analysis of IGR37 cells treated with 3I at different time points. Left Panel. Protein averaged intensity values are reported for lipid transporters (red: SORT1, LRP8, CD36, LDLR, PTGFRN, LRP1, SCARB2, SCARB1, CD63) and PPAR-alpha target genes (grey: LIPE, LIPA, ACSL1, CPT2) in the graph

Among the fatty acids accumulating in IGR37 cells upon BACE2 inhibition, polyunsaturated fatty acids (PUFAs), in particular docosahexaenoic acid (DHA), were especially enriched (Fig. 6A). Many studies indicate that PUFA, including DHA, control gene expression directly stimulating PPARα [43, 44]. When activated by lipid ligands, PPARα translocates to the nucleus and promotes the transcription of genes involved in fatty acid transport and β-oxidation, including ACSL3 and CPT1 [43, 44]. In line with this, we observed elevated expression of canonical PPARα target genes following BACE2 inhibition (Fig. 6B), suggesting that increased lipid uptake activates this lipid-sensing transcriptional program.

To further explore the causal relationship between BACE2 activity, lipid uptake, and PPARα signaling, we performed time-course experiments. Confocal imaging with fluorescent lipid analogues showed that lipid internalization occurs rapidly, within 3 h of BACE2 inhibition (Fig. 6C). DHA uptake from the medium also increased significantly within this timeframe (Fig. 6D, Table S36), and membrane accumulation of lipid transporters was evident at 6 h post-treatment (Fig. 6E). In contrast, transcriptional activation of PPARα target genes was delayed, occurring at later time points (Fig. 6E, Table S37). This temporal separation supports a model in which impaired BACE2-mediated shedding causes membrane accumulation of lipid transporters, thereby enhancing lipid uptake. The resulting intracellular lipid overload, particularly of DHA and other PUFAs, leads to PPARα activation with its downstream effects on lipid metabolism.

### Fatty acid overload impairs cancer cell viability via Lipolysis-Induced stress

To understand how lipid imbalance impacts cell growth, we first examined the effect of intracellular cholesterol accumulation. Since The liver X receptor (LXR) promotes cholesterol efflux and protects against intracellular accumulation [45], we treated IGR37 cells with the synthetic LXR agonist GW3965 to restore cholesterol homeostasis. While GW3965 effectively reduced intracellular cholesterol levels, it failed to rescue cell viability (Fig. S5A, B), suggesting that cholesterol overload is not the primary driver of growth inhibition upon BACE2 loss.

**Fig. 7:**
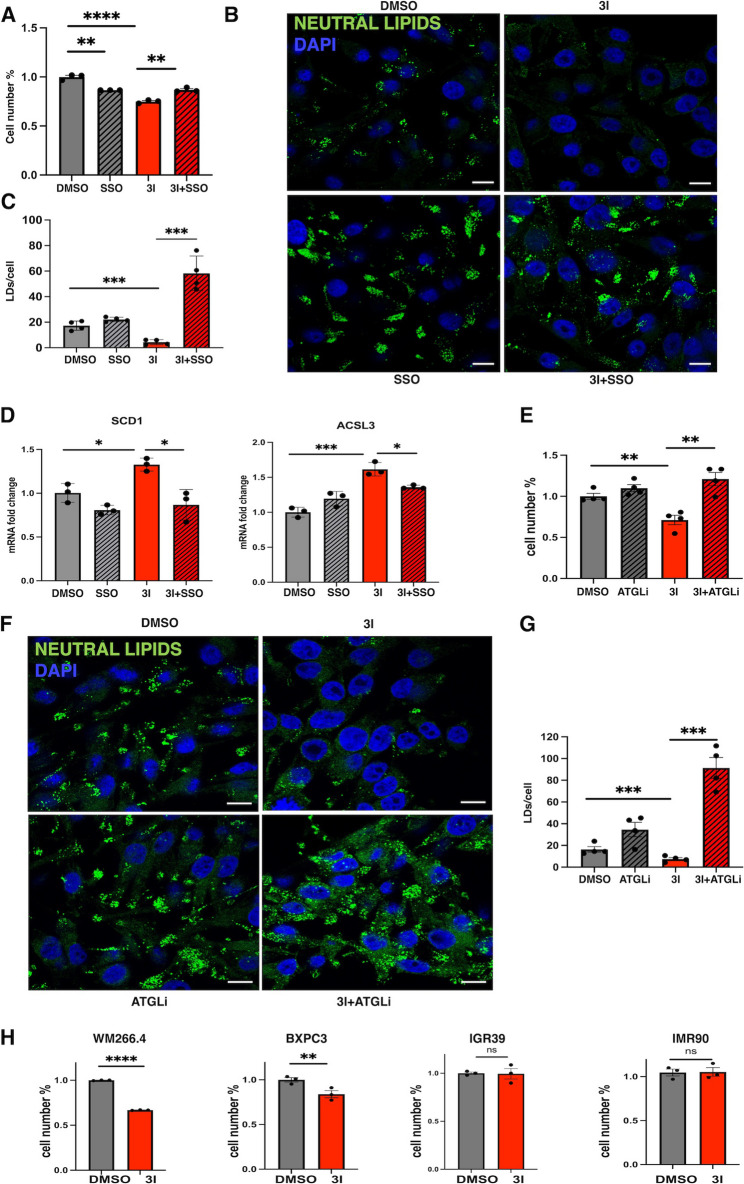
Effect of Lipid Overload on Cell Viability and Lipid Droplets Dynamics.** (A)**. Cell viability analysis assessed by MTT assay. IGR37 cells were treated with DMSO, SSO 150 µM, 3I 5µM and 3I + SSO for 48 h. Data shown are given as mean ± SD. (Student T-test ****palue < 0.0001, **p value < 0.01). *N* = 3. **(B).** Visualization of lipid droplets (LDs) with Nile Red staining in IGR37 cells upon 3I and SSO treatment. A representative fluorescence confocal microscopy image is shown using a double staining of DAPI (blue) as nuclear marker, and Nile Red as LDs marker in green. **(C)**. Quantification of LDs per cell by immunofluorescence analysis using Fiji software. Statistical analysis of LDs per cells was applied for IGR37 cells. (T-test ***p value < 0.001. *N* = 6). Scale bar: 10 μm. **(D)**. PPAR-alpha target genes, ACSL3 and SCD1, mRNA fold change in IGR37 cells treated with DMSO, SSO, 3I and 3I + SSO for 48 h. (Student T-test **p value < 0.01, *p value < 0.05). *N* = 3. **(E)**. Cell viability analysis assessed by MTT assay. IGR37 cells were treated with DMSO, ATGL inhibitor 50µM, 3I 5µM and 3I + SSO for 48 h. Data shown are given as mean ± SD. (Student T-test **p value < 0.01). *N* = 3. **(F)**. Visualization of LDs with Nile Red staining in IGR37 cells upon 3I and ATGL inhibitor 50µM treatment for 48 h. A representative fluorescence confocal microscopy image is shown using a double staining of DAPI (blue) as nuclear marker, and Nile Red as LDs marker in green. **(G)**. Quantification of LDs per cell by immunofluorescence analysis using Fiji software. Statistical analysis of LD per cells was applied for the cell lines reported in the figure. (T-test ***p value < 0.001. *N* = 6). Scale bar: 10 μm. **(H)**. Analysis of cell viability for WM266.4, BXPC3, IGR39 and IMR90 cells (Student T-test ****p value < 0.0001, **p value < 0.001. *N* = 3). WM266.4 cells were treated with 7µM 3I or DMSO for 48 h. BXPC3 cells were treated with 10 µM 3I or DMSO for 48 h, IGR39 were treated with 5µM 3I or DMSO for 48 h, IMR90 cells were treated with 5µM 3I or DMSO for 48 h

We then explored whether fatty acids uptake and accumulation were affecting cell proliferation. Blocking FA uptake (Fig S5C, D) using using Sulfo-N-succinimidyloleate **(**SSO), a fatty acid analogue and irreversible CD36 inhibitor, is sufficient to abolish the cell viability impairment observed in BACE2 inhibited cell (Fig. 7A). These findings indicate that increased FA influx is a major contributor to the anti-proliferative effects of BACE2 inhibition.

Beyond rescuing cell proliferation, SSO treatment also reversed the activation of PPARα target genes (Fig. 7D, S5E, Tables S38–39), confirming that fatty acid overload is the primary driver of PPARα activation and linking extracellular lipid influx to downstream metabolic reprogramming upon BACE2 inhibition.

Interestingly, SSO also restored LDs content in high BACE2 expressing cancer cell (Fig. 7B, C). Time course experiments showed that lipolysis precedes PPARα activation (Fig. S4B, Fig. 6E), prompting us to investigate whether lipolysis contributes to impaired proliferation. We thus inhibited adipose triglyceride lipase (ATGL), a key enzyme in triglyceride breakdown. ATGL inhibition rescued both cell proliferation (Fig. 7E) and LDs integrity (Fig. 7F, G) in BACE2-inhibited cells. These results demonstrate that lipolysis is sufficient to drive metabolic stress and compromise cancer cells survival.

Collectively, our data show that lipid overload impairs cell proliferation primarily through lipolysis-induced metabolic stress, particularly in high-LDs-content cancer cells such as IGR37, WM266.4, and BXPC3. In cells with low basal lipid storage (e.g., IGR39 and IMR90 fibroblasts), BACE2 inhibition promotes LDs formation rather than lipolysis, and cell viability is maintained (Fig. 7H). Notably, in these cells, differently from what observed for IGR37 cells, co-treatment of BACE2 inhibition (3I) with fatty acid uptake inhibition (SSO) did not show any additional effect compared to SSO alone (Fig. S5G), further suggesting that lipid-induced decreased proliferation is specific for high-LDs-content cancer cells.

These results not only highlight BACE2 as a central modulator of lipid flux and storage but also point to a vulnerability in lipid-addicted tumors that could be therapeutically exploited by targeting BACE2 or its downstream lipid-handling pathways.

## Discussion

Our work reveals a previously unrecognized role of BACE2 as a key regulator of lipid metabolism. By studying melanoma and pancreatic cancer cells, we have demonstrated that BACE2 mantains LDs homeostasis by fine-tuning lipid uptake via the shedding of lipid transporters. These findings suggest that BACE2 sustains tumor growth and survival in a lipid-rich environment, positioning it as a central player in cancer metabolic reprogramming [46, 47].

In detail, we showed that BACE2 expression is elevated in proliferative cancer cells with high lipid metabolic activity and abundant LDs content. Given the known function of LDs in sustaining proliferation, survival, metastasis, and therapy resistance [48], our data highlight the role of BACE2 in maintaining this metabolic advantage. Despite growing interest in LDs metabolism as a therapeutic target, the upstream regulatory mechanisms controlling lipid uptake and storage remain unclear [49]. Our results demonstrate that BACE2 modulates the membrane availability of lipid transporters, including LDLR and CD36, via extracellular domain cleavage, thereby controlling lipid influx and LDs stability.

We have elucidated the molecular mechanism by which BACE2 regulates lipid transport proteins using spatial proteomics and mass spectrometry-based peptide mapping. Our analyses revealed that BACE2 cleaves the extracellular domains of lipid transporters. This is supported by the enrichment of peptides from the cytosolic domains exclusively in the membrane fraction following BACE2 inhibition, along with the identification of novel N-terminal cleavage peptides in the secretome of BACE2-proficient cells (Fig. 2). We further show that the tetraspanin CD63, a key organizer of endosomal microdomains, is essential for this process. CD63 is known to facilitate PMEL cleavage by BACE2 in melanosomes [39]. Here, we extend this principle by showing that CD63 is required for BACE2-dependent shedding of multiple substrates, including lipid transporters. CD63 silencing in melanoma cells reduces secretion and increases intracellular accumulation of transporters such as LDLR and CD36, phenocopying BACE2 inhibition. Moreover, BACE2 overexpression fails to promote the shedding of its substrates in the absence of CD63. These results demonstrate that CD63 is essential for positioning BACE2 with its substrates within specific vesicular domains, thereby enabling their cleavage.

The role of CD63 in lipid transporter processing is consistent with its emerging function in cholesterol handling. Previous studies show that CD63 promotes cholesterol transfer within multivesicular bodies (MVBs), enriching intraluminal vesicles (ILVs) with cholesterol [50, 51]. By organizing cholesterol-rich microdomains, CD63 likely creates the physical environment required for efficient BACE2–substrate encounters. The exact determinants of BACE2 substrate selectivity within ILVs remain an important question for future investigation.

We also show that BACE2 expression responds to extracellular lipid availability, suggesting a feedback mechanism whereby BACE2 acts as a lipid sensor, enabling tumor cells to adapt lipid uptake to environmental conditions. BACE2 thus functions as a gatekeeper of lipid transporters, modulating LDs dynamics according to metabolic demands.

**Fig. 8:**
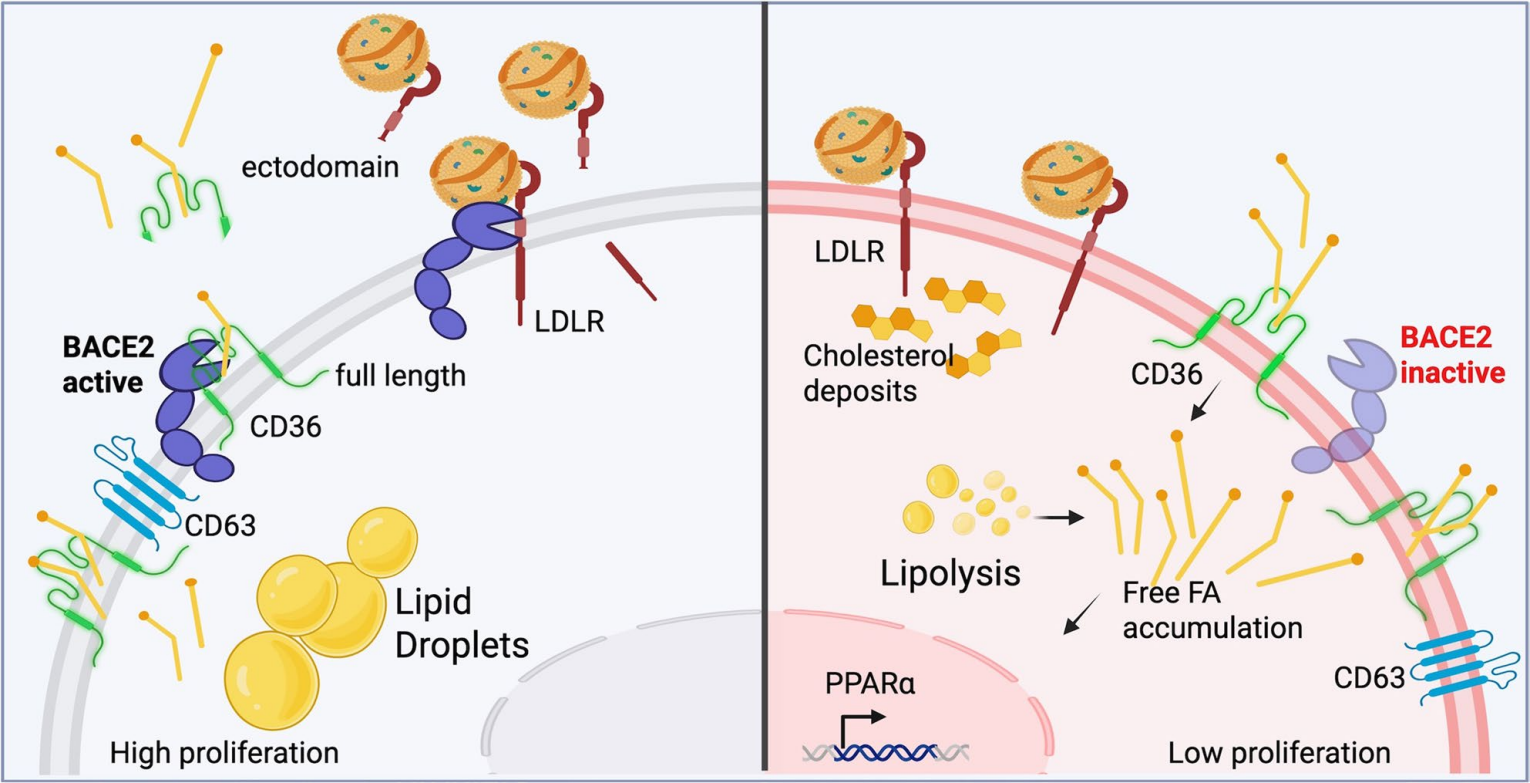
BACE2-Dependent Regulation of lipid metabolism in Cancer Cells. Left panel: BACE2 modulates lipid transporters activity acting as sheddase. This function is facilitated by CD63 microdomain organization. By modulation lipid influx, BACE2 activity supports LDs stability in cancer cells with enhanced lipid metabolism. Right panel: upon BACE2 inhibition, the amount of lipid transporters on the plasma membrane increases, enhancing lipid influx. The resulting accumulation of intracellular cholesterol and FAs exceed the LDs buffering capacity, triggering lipolysis and causing metabolic stress. The excess FAs are detected by PPAR-alpha, which initiates metabolic reprogramming to mitigate lipid overload

Upon BACE2 inhibition, we observe an increase in lipid uptake, due to membrane accumulation of lipid transporters. This leads to alterations in intracellular fatty acid (FA), especially DHA, and cholesterol levels. Cells with low basal LDs content buffer excess lipids in newly formed LDs, thus preserving viability. In contrast, lipid-rich cells experience lipid overload, which triggers ATGL mediated lipolysis, resulting in cellular dysfunction and impaired proliferation. The accumulation of FAs triggers the activation of the PPARα transcriptional program to rebalance lipid metabolism (Fig. 8). This transcriptional response occurs downstream of lipid uptake and lipolysis, indicating that the primary driver of metabolic stress is uncontrolled lipid turnover, not initial changes in gene expression. However, the precise mechanism by which lipid overload initiates lipolysis and the link with PPARα activation remain to be elucidated.

Mechanistically, we show that blocking either fatty acid uptake using a CD36 inhibitor or lipolysis via ATGL inhibition, restores LDs integrity and rescues cell viability in BACE2-deficient cells. These findings confirm that lipolysis-induced stress, rather than canonical apoptosis or necrosis, is a key driver of growth arrest under BACE2-deficient conditions. Supporting this, our prior study [21], including Annexin V/PI staining, showed no significant increase in apoptosis or necrosis upon BACE2 inhibition. Furthermore, cell cycle analyses in the same study revealed a proliferative delay marked by G1 or G2 phase accumulation, depending on the cell type, which is likely a consequence of the observed lipid metabolic imbalance. Future work will investigate the possible involvement of lipid peroxidation and ferroptotic pathways in mediating this metabolic stress.

The observation that DHA and other PUFAs accumulate following BACE2 inhibition opens new therapeutic avenues. Recent studies show that excess PUFAs, especially under acidic tumor microenvironmental conditions, promote lipid peroxidation and ferroptosis in cancer cells [52]. Here, the inability to store lipids in LDs increases susceptibility to oxidative lipid damage, suggesting that BACE2 inhibition in combination with dietary PUFA enrichment could amplify lipotoxicity and enhance anticancer efficacy.

Our results therefore suggest that BACE2 inhibition could be a promising strategy to disrupt lipid metabolism and inducing lipotoxicity in lipid-dependent cancer cells. However, the effects are context-dependent and will vary depending on factors like initial lipid stores and metabolic demands of the tumor. Preclinical efforts targeting lipid metabolism, such as inhibitors of fatty acid synthase (FASN), are already progressing into clinical stages [53], while new combined therapeutic strategies including targeting fatty acid uptake or lipid desaturation are under investigation [53]. Our findings may provide a complementary avenue by targeting lipid uptake regulation.

Although our study is limited to in vitro models, the connection between BACE2 and lipid metabolism is supported by in vivo evidence. In BACE2 knockout (BKO) mice fed a high-fat diet there is increased body weight, food intake, and hepatic lipid droplet accumulation, along with hyperinsulinemia, suggesting that the mechanisms described in our work are conserved in vivo [54]. A recent xenograft study demonstrated that BACE2 overexpression promotes liver tumour growth and increased tumour weight [22]. This study also revealed that ENO1-BACE2 axis promote LDLR cleavage, further sustaing the involvement of BACE2 in lipid regulation. Additional in vivo studies showed that silencing BACE2 significantly reduces tumor volume and weight in ocular melanoma xenografts [55], moreover pharmacological BACE inhibition decreases tumor burden in skin cancer models [56]. These in vivo data align with our proposed model and underscore BACE2’s therapeutic potential. Indeed, dual BACE1/BACE2 inhibitors show good tolerability in mice, with only hair depigmentation, as consequence of BACE2’s role in melanosomes, and without systemic toxicity [57]. Furthermore, BACE2-null mice are viable, fertile, and display no major phenotypic defects [58], suggesting a favorable therapeutic window.

## Conclusions

In conclusion, we demonstrated that BACE2 regulates lipid homeostasis, prevents lipid overload, and supports the needs of rapidly proliferating cells. By elucidating BACE2’s regulatory role in lipid metabolism, this study provides insights into its potential as a therapeutic target for cancers exhibiting high lipid dependency. This work not only advances our understanding of BACE2’s non-amyloidogenic functions, but also reveals novel metabolic vulnerabilities in cancer cells that could be leveraged to inhibit tumor growth.

## Supplementary Information

Supplementary Material 1.

Supplementary Material 2.

Supplementary Material 3.

Supplementary Material 4.

Supplementary Material 5.

Supplementary Material 6.

Supplementary Material 7.

Supplementary Material 8.

## Data Availability

The mass spectrometry proteomics data have been deposited to the ProteomeXchange Consortium via the PRIDE [59] partner repository with the dataset identifiers: PXD058702, PXD058731, PXD058751, PXD058789.Lipidomics data have been deposited to the EMBL-EBI MetaboLights database with the identifier MTBLS11863.

## Abbreviations

BACE2: Beta-site APP-cleaving enzyme 2
PDAC: Pancreatic Ductal Adenocarcinoma
TCGA: The Cancer Genome Atlas
GTEx: Genotype-Tissue Expression
GEPIA: Gene Expression Profiling Interactive Analysis
CCLE: Cancer Cell Line Encyclopedia
LD: Lipid Droplet
TAG: Triacylglycerol
SM: Sphingomyelin
ST: Sterol
LION: Lipid Ontology
BACE2i (3I): BACE2 Inhibitor (compound 3I)
LDLR: Low-Density Lipoprotein Receptor
CD36: Cluster of Differentiation 36 (Fatty Acid Translocase)
SCARB1: Scavenger Receptor Class B Member 1
SORT1: Sortilin 1
LRP1: LDL Receptor Related Protein 1
LRP8: LDL Receptor Related Protein 8
APP: Amyloid Precursor Protein
PMEL: Premelanosome Protein
IGF2R: Insulin-like Growth Factor 2 Receptor
N-TAILS: N-terminal Amine Isotopic Labeling of Substrates
DHA: Docosahexaenoic Acid
PUFA: Polyunsaturated Fatty Acids
FA: Fatty Acid
PPARα: Peroxisome Proliferator-Activated Receptor Alpha
ACLY: ATP Citrate Lyase
ACACA: Acetyl-CoA Carboxylase Alpha
ACSL3: Acyl-CoA Synthetase Long-Chain Family Member 3
FASN: Fatty Acid Synthase
CPT1 / CPT2: Carnitine Palmitoyltransferase 1 / 2
LIPE: Hormone-Sensitive Lipase (Lipase E)
ATGL: Adipose Triglyceride Lipase
ACAT2: Acetyl-CoA Acetyltransferase 2
ACSS2: Acyl-CoA Synthetase Short Chain Family Member 2
CYP51A1: Cytochrome P450 Family 51 Subfamily A Member 1
DHCR24: 24-Dehydrocholesterol Reductase
FDFT1: Farnesyl-Diphosphate Farnesyltransferase 1
LSS: Lanosterol Synthase
MVD: Mevalonate Diphosphate Decarboxylase
RETSAT: Retinol Saturase
SSO: Sulfo-N-succinimidyloleate (CD36 inhibitor)
LXR: Liver X Receptor
GW3965: Synthetic LXR Agonist
CCLE: Cancer Cell Line Encyclopedia
IRM90: Human Lung Fibroblast Cell Line
IGR37 / IGR39: Melanoma Cell Lines (High / Low BACE2)
WM266.4 / WM115: Melanoma Cell Lines (High / Low BACE2)
BXPC3 / CAPAN2: Pancreatic Cancer Cell Lines
HEK293T: Human Embryonic Kidney 293T Cells
