# Supplementary figures and images for: BACE2 tunes lipid uptake through lipid transporters shedding supporting cancer cell proliferation
Source: J Exp Clin Cancer Res. 2026 Jan 8;45:36. doi: 10.1186/s13046-025-03626-x (PMC12870435; doi:10.1186/s13046-025-03626-x)

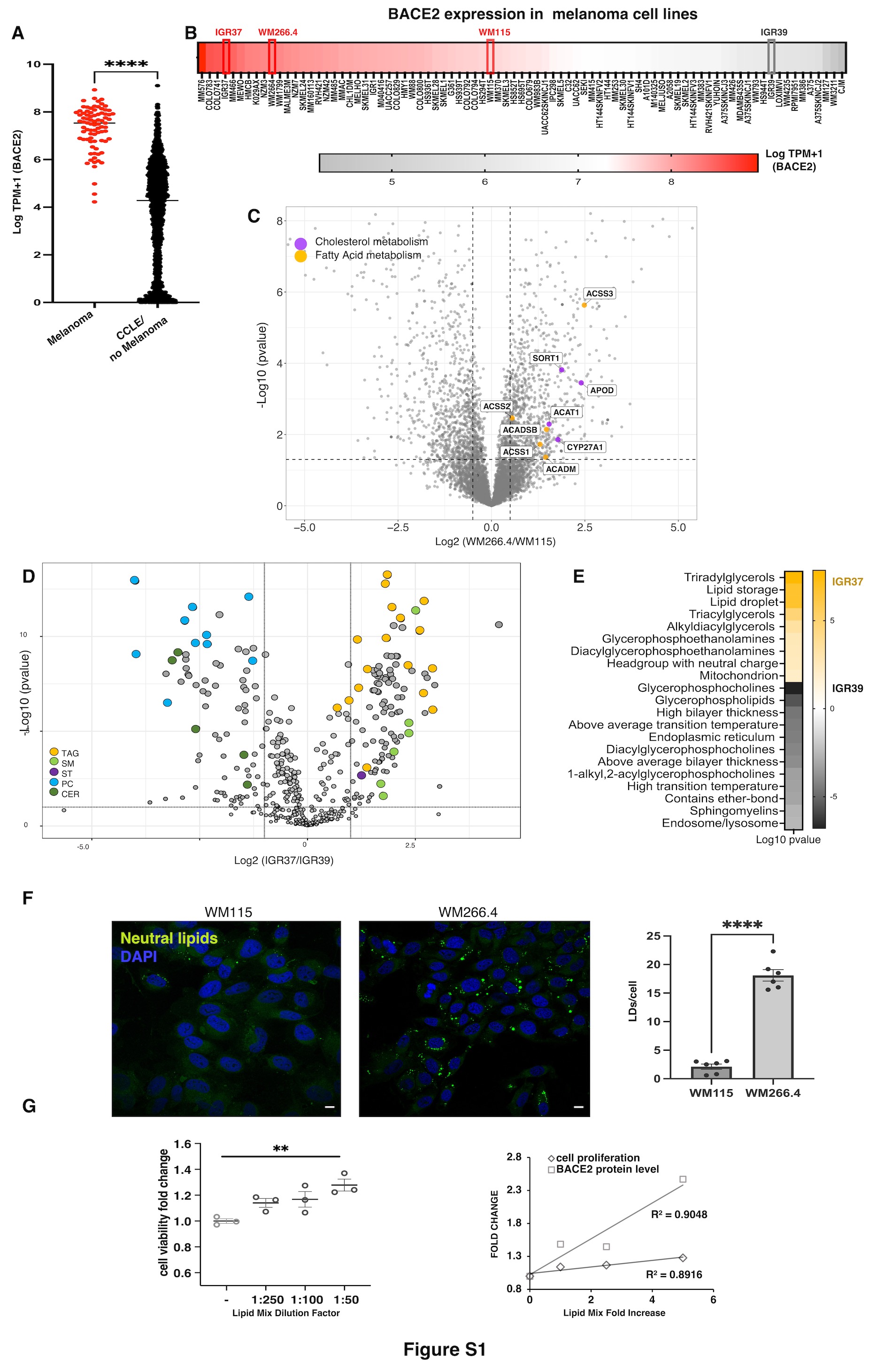

Supplement: Supplementary file 1 — Supplementary Material 1. [file 13046_2025_3626_MOESM1_ESM.jpg]

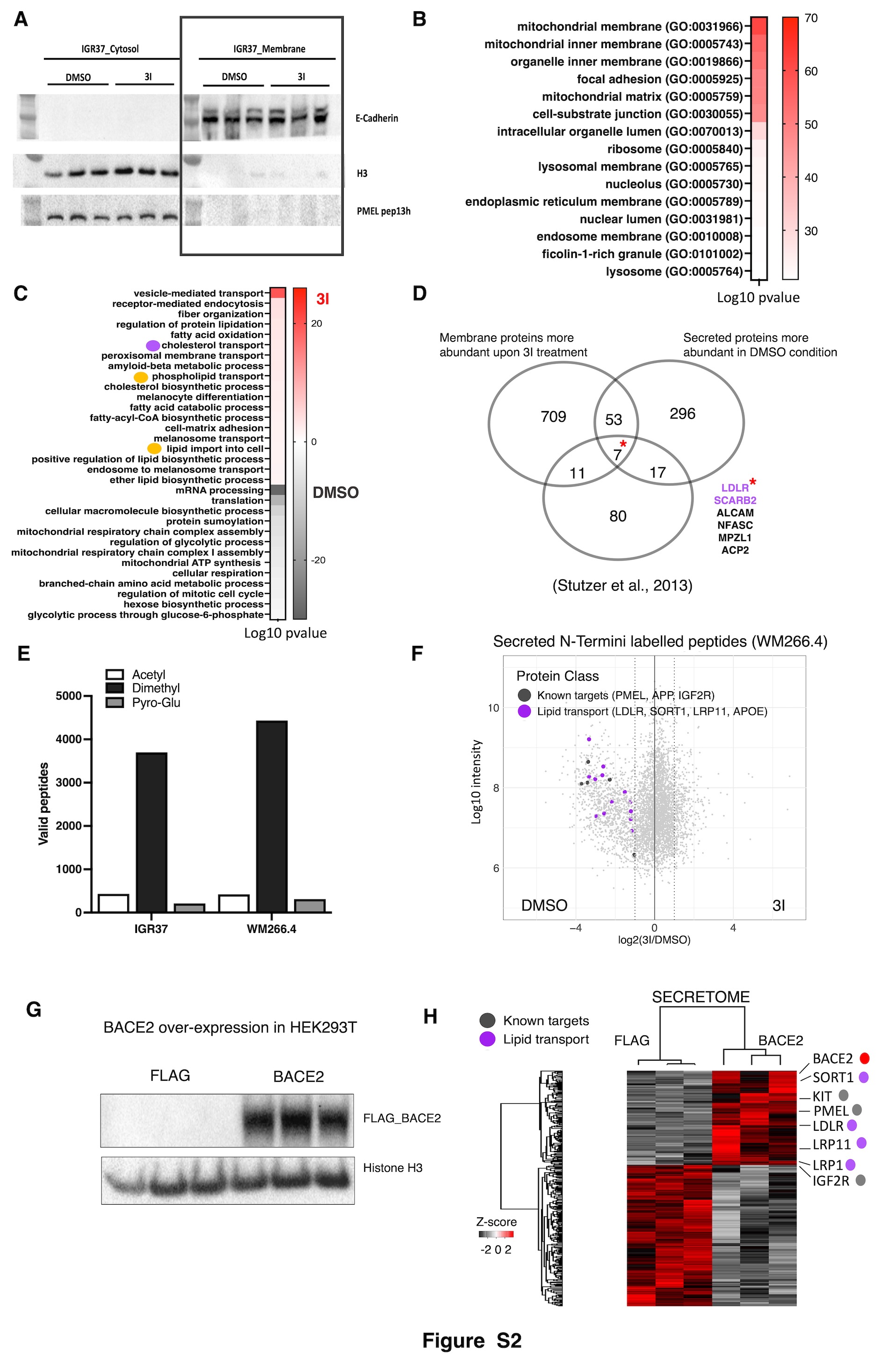

Supplement: Supplementary file 2 — Supplementary Material 2. [file 13046_2025_3626_MOESM2_ESM.jpg]

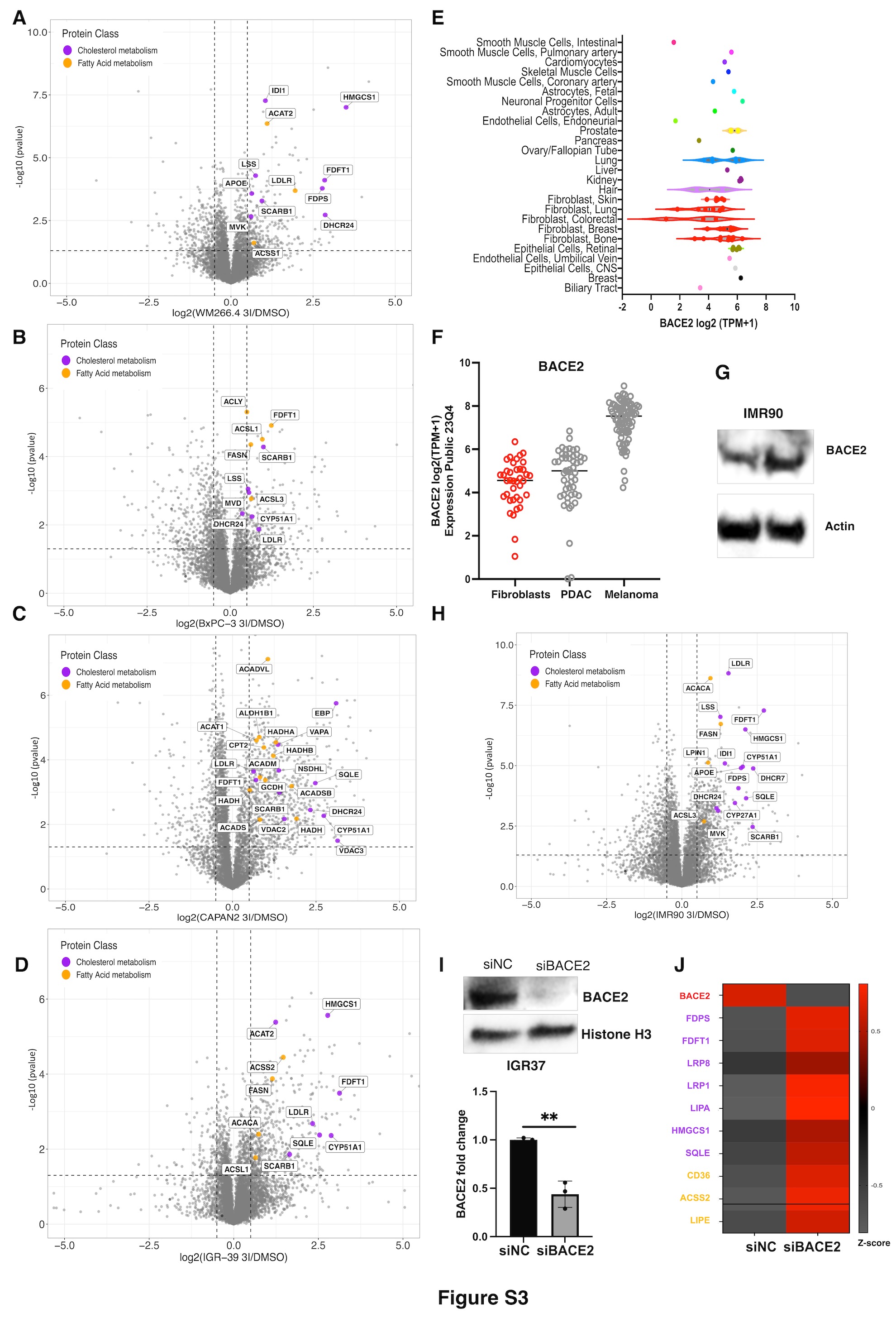

Supplement: Supplementary file 3 — Supplementary Material 3. [file 13046_2025_3626_MOESM3_ESM.jpg]

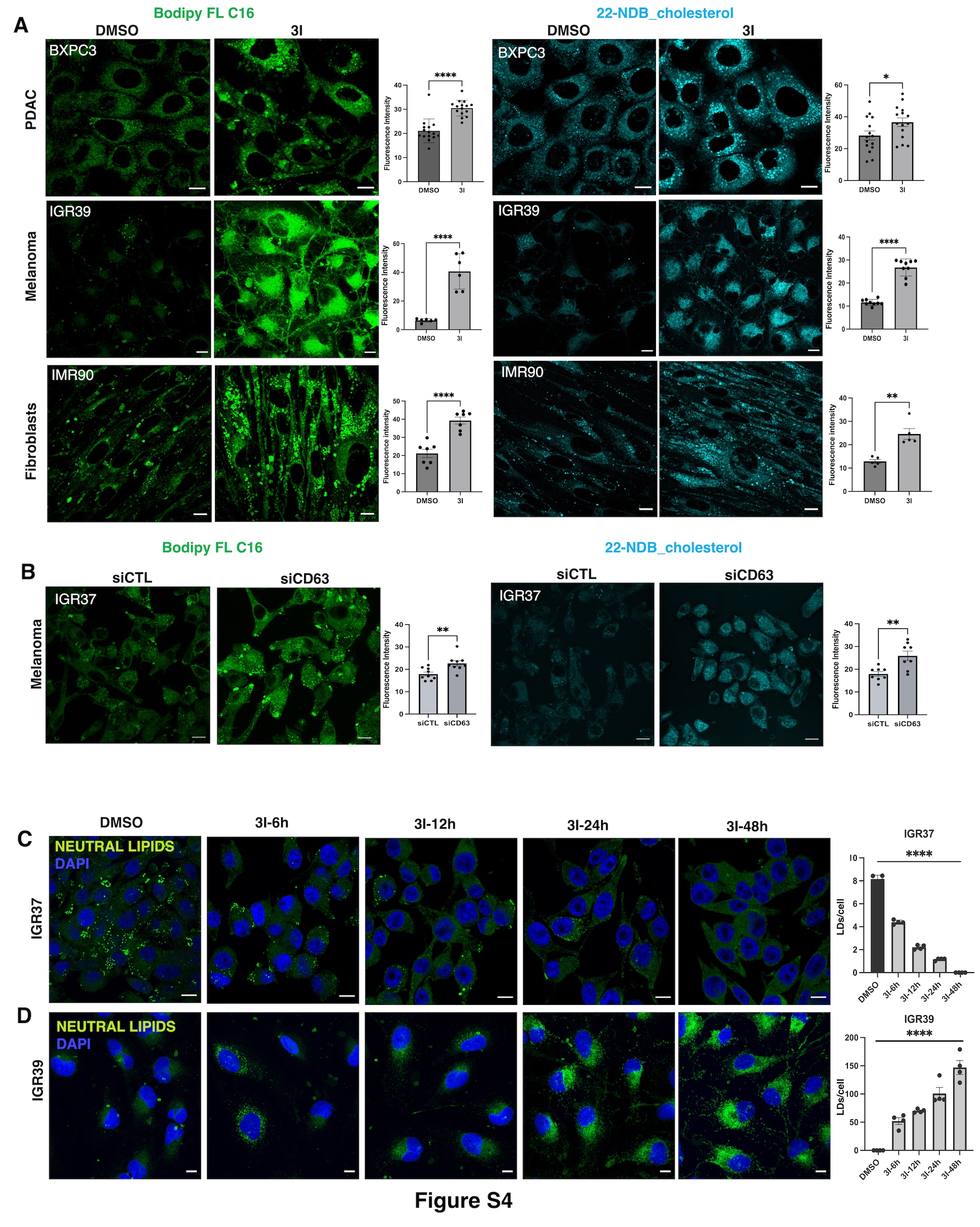

Supplement: Supplementary file 4 — Supplementary Material 4. [file 13046_2025_3626_MOESM4_ESM.jpg]

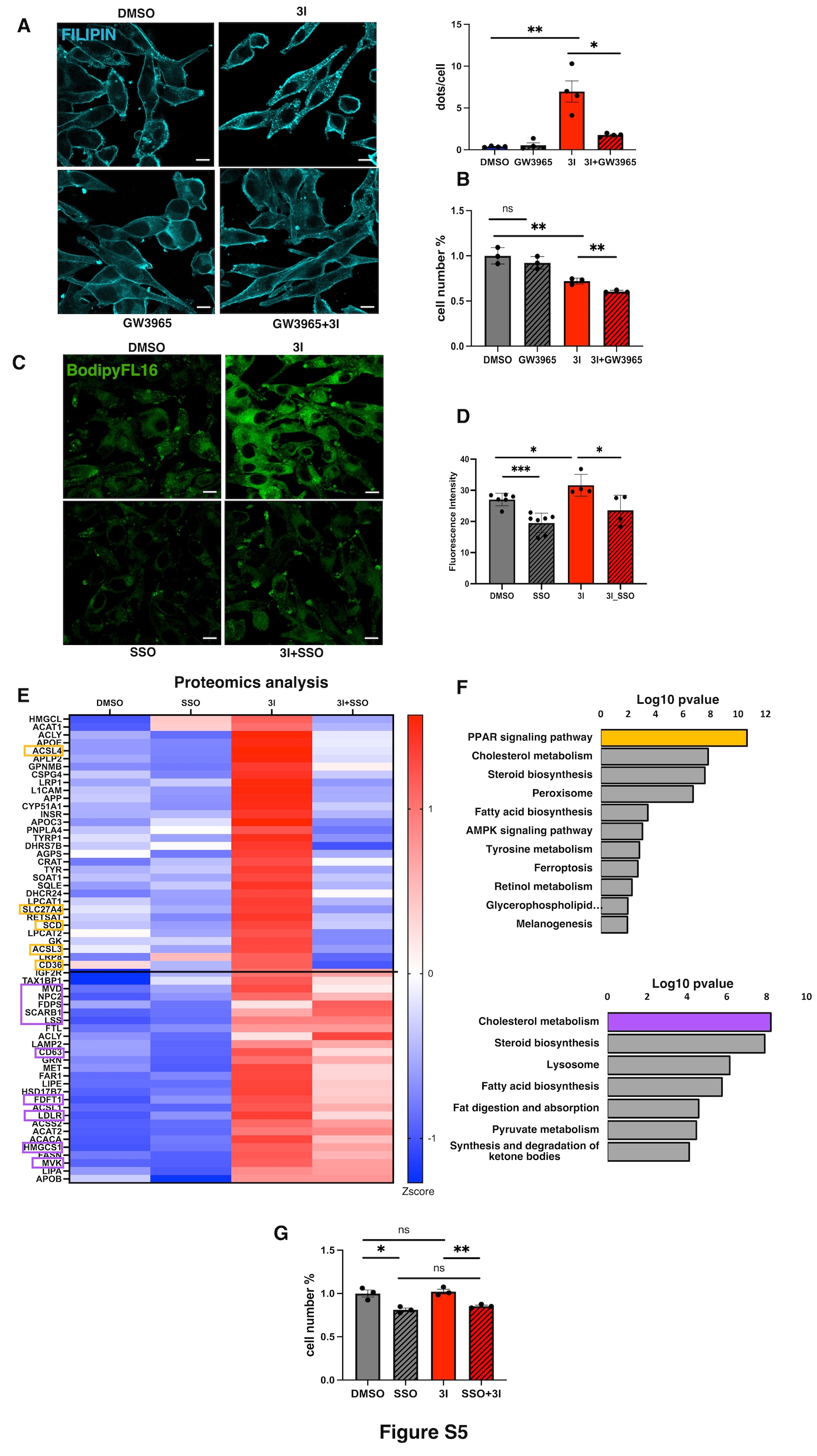

Supplement: Supplementary file 5 — Supplementary Material 5. [file 13046_2025_3626_MOESM5_ESM.jpg]
